# Separation of timescales for the seed bank diffusion and its jump-diffusion limit

**DOI:** 10.1007/s00285-021-01596-0

**Published:** 2021-04-28

**Authors:** Jochen Blath, Eugenio Buzzoni, Adrián González Casanova, Maite Wilke Berenguer

**Affiliations:** 1 Institut für Mathematik, Technische Universität Berlin, Berlin, Germany; 2 Instituto de Matemáticas, Universidad Nacional Autónoma de México, Mexico City, Mexico; 3 Fakultät für Mathematik, Ruhr-Universität Bochum, Bochum, Germany

**Keywords:** Strong seed bank, Two-island model, Separation of timescales, Diffusion limits, Jump-diffusion, Duality, Primary  60K35, Secondary  92D10

## Abstract

We investigate scaling limits of the seed bank model when migration (to and from the seed bank) is ‘slow’ compared to reproduction. This is motivated by models for bacterial dormancy, where periods of dormancy can be orders of magnitude larger than reproductive times. Speeding up time, we encounter a separation of timescales phenomenon which leads to mathematically interesting observations, in particular providing a prototypical example where the scaling limit of a continuous diffusion will be a jump diffusion. For this situation, standard convergence results typically fail. While such a situation could in principle be attacked by the sophisticated analytical scheme of Kurtz (J Funct Anal 12:55–67, 1973), this will require significant technical efforts. Instead, in our situation, we are able to identify and explicitly characterise a well-defined limit via duality in a surprisingly non-technical way. Indeed, we show that moment duality is in a suitable sense stable under passage to the limit and allows a direct and intuitive identification of the limiting semi-group while at the same time providing a probabilistic interpretation of the model. We also obtain a general convergence strategy for continuous-time Markov chains in a separation of timescales regime, which is of independent interest.

## Motivation and main results

In this extended introductory section, we first provide some background on the biological concept of dormancy and its relevance in particular in microbial communities. This is followed by a short review of modelling approaches for dormancy in population genetics, where we think that dormancy might be seen as an additional evolutionary force, interacting with other forces such as genetic drift in complex ways. Since dormancy periods vary over several orders of magnitude (depending on the underlying species and environmental conditions), we aim for a systematic classification of relevant timescales, leading to the distinction of three separate scaling regimes. While the first two regimes have been modelled and analysed in population genetics before, the last one, leading to a separation of timescales between genetic drift and dormancy periods, is new, and completes the picture (at least on the level of ‘toy models’) of modelling scenarios. Our results for this regime will be presented in this introduction both for the forward-in time population model as well as for the dual genealogical processes, leading to novel scaling limits, which are interesting also from a purely mathematical perspective.

The proofs of these results can be found in Sects. 2 and 3 for the results going backwards and forwards in time, respectively. We believe that our rather direct method of proof to obtain and characterise these limits, making extensive use of duality for Markov processes, can be applied in a variety of situations, so that in each section, we first present the corresponding methodology in a general set-up and then discuss its application to our concrete motivation.

*Background on dormancy* Dormancy is a complex trait that has developed independently in many species across the tree of life and comes in many different guises. Originally, theory for dormancy and the resulting seed banks has be developed in the context of bet-hedging strategies for plants Cohen (1966). However, dormancy is also a highly common trait in microbial communities, with important consequences for their evolutionary, ecological and pathogenic properties.

Here, we define dormancy as the ability of (micro-) organisms to enter and leave a state of vanishing metabolic activity. It has been observed for many habitats that at any given time a large fraction of micro-organisms can be in such a dormant state. For example, more than $80 \%$ of bacteria in soil are reported to be metabolically inactive, forming large ‘seed banks’ comprised of dormant individuals, see Lennon and Jone (2011). While dormancy seems to be an efficient and wide-spread strategy, e.g. to withstand unfavourable environmental conditions, competitive pressure, or antibiotic treatment, it is at the same time a costly trait whose maintenance involves energy and a sophisticated ‘switching machinery’.

Dormancy also plays a role in various (human) diseases. So-called *persister cells*, that may evade antibiotic treatment by remaining in a state of low activity, play a major role in chronic infections, cf. Fisher et al. (2017), and individual cell dormancy is linked to relapses in cancer, cf. Marx (2018), Endo and Inoue (2019).

In this paper, we will focus on *microbial seed banks*. Lennon and Jone (2011) and Shoemaker and Lennon (2018) provide a broad overview of this rich and fascinating field and serve as a motivation in the present paper. Given the relevance of biological systems exhibiting dormancy, investigating the mathematical implications of dormancy in large populations seems to be a timely and interesting task.

*Classification of the duration of dormancy: Known models and motivation for this paper* As indicated above, dormancy comes in many different forms, specific to the involved species and environments. One variation lies in the duration of dormancy periods: While in some microbial species dormancy periods last at most a few days, others stay dormant for prolonged periods of time, and some, e.g. bacterial endospores, have been reported to successfully resuscitate from dormancy after millions of years (Shoemaker and Lennon 2018; Cano and Borucki 1995; Johnson et al. 2007; Morono et al. 2020). The theoretical derivation and analysis of mathematical models may help to identify, understand and classify the different effects of dormancy, on suitable timescales, on the population dynamics and genealogical processes of the underlying populations.

Hence, in this paper, we consider the consequences of dormancy and seed banks in the framework of *population genetics*. More precisely, we are interested in the interplay of dormancy and the classical evolutionary force of *random genetic drift*, in particular with respect to its sensitivity to the duration of dormancy periods.

In a bi-allelic, haploid population that reproduces according to the Wright-Fisher model, the frequency of a given allele converges to the *Wright-Fisher diffusion*, given as the solution to

$$\begin{aligned} \mathrm{d}Z(t) = \sqrt{Z(t)(1-Z(t))} \mathrm{d}B(t), \end{aligned}$$

where $(B(t))_{t\ge 0}$ is a standard Brownian motion, if one measures time in the *coalescent timescale* (also known as the *evolutionary timescale*), i.e. on the order of the population size as this tends to infinity. This diffusion is dual to the *block-counting process of the Kingman coalescent* which in turn describes the genealogy of the population. These objects serve as a reference for populations without dormancy and are widely studied and applied in biology and mathematics alike. See e.g. Wakeley (2009) or Etheridge (2011) for an overview. We will consider suitable extensions incorporating dormancy.

We propose to distinguish three regimes comparing the duration of dormancy periods to the coalescent timescale, i.e. the scale at which the random genetic drift acts.

1. *Dormancy periods are small compared to the coalescent timescale.*

**Fig. 1:**
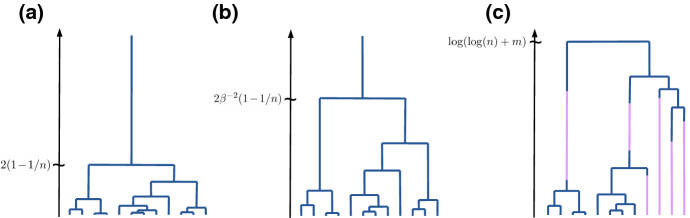
Typical realisations of **a** the Kingman coalescent, where lineages merge at rate 1 per pair, **b** a delayed Kingman coalescent, where lineages merge at rate $\beta ^{2}<1$ per pair, and **c** the seed bank coalescent, see Def. 1.2. In the seed bank coalescent there are two kinds of lines: blue lines are active lineages, while purple lines are dormant lineages. The differences can be seen in the (asymptotic) *expected* time to the most recent ancestor when started with a sample of *n* (active and *m* dormant) individuals given on the time-axis (colour figure online)

**Fig. 2:**
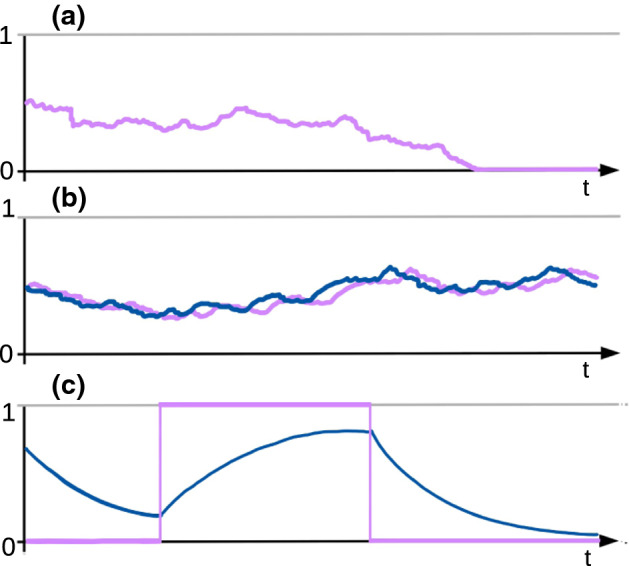
Typical realisations of the trajectory of **a** a time-changed Wright-Fisher diffusion, where the time-change is an effect of a weak seed bank, **b** the seed bank diffusion, with the frequency of a given allele in the active population displayed in blue and in the dormant population, in purple, **c** the frequency process $(\tilde{X}(t),\tilde{Y}(t))$, using the same colour code (colour figure online)

In 2001, Kaj et al. (2001) introduced a model for dormancy in the following fashion: instead of always choosing the ancestor in the preceding generations like in the Wright-Fisher model, individuals are allowed to choose an ancestor several generations in the past. Their lineages thus ‘jump’ this number of generations and can be interpreted as dormant during that time. If we denote by $B\ge 1$ the expected size of the ‘jump’, the genealogy of the model converges on the coalescent timescale to a *delayed Kingman coalescent*, depicted in Fig. 1b, where coalescences occur at rate $\beta ^2$, where $\beta :=1/B$, instead of at rate 1, cf. Kaj et al. (2001), Blath et al. (2013). This in turn is dual to the *delayed Wright-Fisher diffusion*

1 $$\begin{aligned} \mathrm{d}\tilde{Z}(t) = \sqrt{\beta ^2 \tilde{Z}(t)(1-\tilde{Z}(t))} \mathrm{d}B(t), \end{aligned}$$

that again describes the frequency of a given allele in the population, cf. Fig. 2a. Note that $\beta$ does not depend on the population size, whence its qualitatively weak impact on the coalescent timescale.

2. *Dormancy periods on the order of the coalescent timescale*

For microbial species, however, dormancy times can be much longer than just a few ‘generations’, In this set-up, Lennon and Jone (2011) proposed a model based on two reservoirs, the ‘active’ and the ‘dormant’ population, between which individuals ‘migrate/switch’ via initiation of and resuscitation from dormancy, at fixed rates. A mathematical model for ‘spontaneous/stochastic’ switching (observed in nature under stable environmental conditions, cf. Epstein 2009; Shoemaker and Lennon 2018), was introduced and studied in Blath et al. (2016). This is reminiscent of the ‘two-island model’ (Wright 1931; Moran 1959) with the notable difference of the absence of reproduction on the second island.

If the size of the active and dormant population are proportional with the ratio given by some $K>0$, the frequencies *X*(*t*) and *Y*(*t*) of a given allele in the active and dormant population, respectively, when time is measured on the coalescent timescale, are described by the *seed bank diffusion*, cf. Fig. 2b. This diffusion was first introduced in Corollary 2.5 in Blath et al. (2016). The existence of a unique strong solution that is Feller follows from Theorem 3.2 and Remark 3.2 in Shiga and Shimizu (1980), see also Greven et al. (2020) for a more general seed bank diffusion.

### Definition 1.1

(*Seed bank diffusion*) Let $(B(t))_{t\ge 0}$ be a standard Brownian motion and *c*, *K* finite positive constants. The $[0,1]^2$-valued continuous strong Markov process $(X(t), Y(t))_{t \ge 0}$ given as the unique strong solution of the initial value problem

2 $$\begin{aligned} {\left\{ \begin{array}{ll} \mathrm{d} X(t) = c(Y(t) -X(t))\mathrm{d}t + \sqrt{X(t)(1-X(t))}\mathrm{d}B(t), \\ [.1cm] \mathrm{d} Y(t) = Kc(X(t) -Y(t))\mathrm{d}t, \end{array}\right. } \end{aligned}$$

with $(X(0), Y(0)) =(x,y) \in [0,1]^2$, is called *seed bank diffusion* with parameters *c*, *K*, starting at $(x, y)\in [0,1]^2$.

The genealogy of such a population is given by the *seed bank coalescent*, introduced in Definition 3.2 in Blath et al. (2016). Here, lineages can switch between an active and a dormant state independently (hence ‘spontaneous’ switching) at a given rate $c>0$. While the active lineages behave like the Kingman coalescent, dormant lineages are prohibited from coalescing, as depicted in Fig. 1c.

That dormancy appears in such a prominent form in the coalescent and in the diffusion and therefore is visible on the coalescent timescale is due to the underlying scaling assumptions of the model. These imply that dormancy times are of the order of the population size and therefore on the coalescent timescale. Here, many population genetic quantities and statistics are affected in non-trivial ways, see Blath et al. (2015), Blath et al. (2016) and Blath et al. (2020b) for a discussion of the scaling assumptions and further extensions of the model. Since the seed bank here has a major qualitative effect on both the diffusion and the coalescent, this is sometimes referred to as the *strong seed bank model*.

As in the previous models, an important mathematical tool in our analysis will be the formal duality relation between the seed bank diffusion $(X(t),Y(t))_{t \ge 0}$ and the *block-counting process of the seed bank coalescent* $(N(t),M(t))_{t \ge 0}$. Note that the notion of a ‘block’ comes from the mathematical definition of a coalescent as a partition-valued process. In the biological context, the process could as well be denoted the *line-counting process*, keeping track of the number of ancestral lines presents at each time in the past.

### Definition 1.2

(*Block-counting process of the seed bank coalescent*) Let $E := {\mathbb {N}}_0\times {\mathbb {N}}_0$. Let $c,K>0$. We define $(N(t),M(t))_{t\ge 0}$ to be the continuous-time Markov chain taking values in *E* with conservative *Q*-matrix *R* given by

3 $$\begin{aligned} R_{(n,m), ({\bar{n}},{\bar{m}})} = {\left\{ \begin{array}{ll} \left( {\begin{array}{c}n\\ 2\end{array}}\right) &{} \text {if } ({\bar{n}},{\bar{m}}) = (n-1,m),\\ cn &{} \text {if } ({\bar{n}},{\bar{m}}) = (n-1,m+1),\\ cKm &{} \text {if } ({\bar{n}},{\bar{m}}) = (n+1,m-1),\\ 1 - \left( {\begin{array}{c}n\\ 2\end{array}}\right) - cn - cKm, &{} \text {if } ({\bar{n}},{\bar{m}}) = (n,m),\\ 0, &{} \text {otherwise.} \end{array}\right. } \end{aligned}$$

This continuous-time Markov chain introduced in Definition 2.7 in Blath et al. (2016), satisfies the moment duality

4 $$\begin{aligned} \mathbb {E}^{x,y}\big [X(t)^nY(t)^m\big ]= \mathbb {E}_{n,m}\big [x^{N(t)}y^{M(t)}\big ] \end{aligned}$$

for every $t>0$, for every $(x,y)\in [0,1]$ and for every $n,m\in \mathbb {N}_0$, see Theorem 2.8 in Blath et al. (2016). In other words, the distribution of the seed bank diffusion at any time *t* is uniquely determined by the moment dual at said time.

3. *Dormancy periods are large compared to the coalescent timescale.*

In view of the (potentially) extreme duration of dormancy times of bacterial spores, it is natural to ask: What happens in the third natural scaling-regime, when dormancy times are long in comparison to the scale on which genetic drift acts? This is the question answered in this manuscript in the following subsections.

To this end, we consider scaling limits of the above seed bank/two-island model when migration between active and dormant states (say at rate *c*) and reproduction (say at rate 1) act on different timescales, that is *c* being much smaller than 1. Interesting limits can only be expected when switching to a ‘fast’ super-evolutionary timescale. Indeed, if one just lets $c \rightarrow 0$, then one obtains the trivial limit where the active population follows a Wright-Fisher diffusion and a Kingman coalescent, respectively, and is completely separated from the dormant population, as can be readily seen from (2) and (3). Hence, in order to capture the effect of long dormancy times one needs to speed up time by a factor 1/*c*, as $c \rightarrow 0$, thus switching to a new timescale, which we will refer to as the super-evolutionary timescale. At this super-evolutionary timescale migration between the active and the dormant population occurs at rate 1 while reproduction, and hence genetic drift, acts ‘instantaneously’. Intuitively, fast reproduction should drive the *X* coordinate of the diffusion process immediately towards the boundaries 0 and 1, which then only rarely switches between these states due to immigration of ‘ancient’ alleles. This is indeed what we will see below.

This scaling regime also leads to mathematically appealing problems. The naïve scaling limit would lead to a coefficient of “$\infty$” for the genetic drift in the seed bank diffusion and an infinite coalescent rate in the seed bank coalescent, respectively, and we thus need to find a way to rigorously identify and describe such a ‘degenerate’ mathematical limit.

*Main results under separation of timescales: the frequency process* The following two theorems provide the main results for the frequency processes of Wright-Fisher models with seed banks, if dormancy times are sufficiently long for the timescales of dormancy and genetic drift to separate. Note that we switch to the super-evolutionary timescale.

### Theorem 1.3

Let $(X^{c}(t), Y^{c} (t))_{t \ge 0}$ be the seed bank diffusion given in Definition 1.1 with migration rate $c>0$. Assume that the initial distributions $(X^{c}(0), Y^{c}(0))$ converge weakly to an $(x,y) \in [0,1]^2$ as $c\rightarrow 0$. Then, there exists a strong Markov process $(\tilde{X}(t),\tilde{Y}(t))_{t\ge 0}$, started in $(\tilde{X}(0),\tilde{Y}(0))=(x,y)$ with the property that for any sequence of migration rates with $c_{\kappa }\rightarrow 0$ when $\kappa \rightarrow \infty$,

$$\begin{aligned} \left( X^{c_{\kappa }}\left( \frac{1}{c_{\kappa }}t\right) , Y^{c_{\kappa }} \left( \frac{1}{c_{\kappa }}t\right) \right) _{t \ge 0} \xrightarrow {\mathrm{f.d.d.}} (\tilde{X}(t),\tilde{Y}(t))_{t\ge 0} \quad \text {as }\kappa \rightarrow \infty . \end{aligned}$$

Furthermore,

5 $$\begin{aligned} \lim _{t\rightarrow 0}\mathbb {P}\big \{\tilde{X}(t)=1\big \}=1-\lim _{t\rightarrow 0}\mathbb {P}\big \{\tilde{X}(t)=0\big \} = x \end{aligned}$$

and we may choose $(\tilde{X}(t),\tilde{Y}(t))_{t\ge 0}$ to be cádlág and such that for every $t>0$ $(\tilde{X}(t),\tilde{Y}(t))\in \{0,1\}\times [0,1]$.

Here, càdlàg stands for *continue à droite, limite à gauche*, i.e. the property of a path to be right-continuous for every $t\ge 0$ and have a limit from the left for every $t>0$.

Note that the above convergence is in the sense of the finite-dimensional distributions (f.d.d.), which uniquely determines the law of the limit. As indicated above, it will have jumps in the first component $\tilde{X}$, which is remarkable since the prelimiting processes all have continuous paths. In order to understand this, we prove in Proposition 3.8 that, if started in $\{0,1\}\times [0,1]$, $(\tilde{X}(t),\tilde{Y}(t))_{t\ge 0}$ coincides in distribution with a Feller process $({\bar{X}}(t),{\bar{Y}}(t))_{t\ge 0}$ taking values in $\{0,1\}\times [0,1]$ which is defined via the generator

6 $$\begin{aligned} \bar{\mathcal A} f(x,y)&= (1-x)y(f(1,y)-f(x,y)) + x(1-y)(f(0,y)-f(x,y))\nonumber \\&\qquad + K(x-y)\frac{\partial f}{\partial y} (x,y), \end{aligned}$$

for functions *f* in $\{f: \{0,1\}\times [0,1]\rightarrow \mathbb {R}\mid f(0,\cdot ), f(1,\cdot ) \in \mathcal C^1([0,1],\mathbb {R})\}$.

The dynamics of the process $(\tilde{X}(t), \tilde{Y}(t))_{t \ge 0}$ are therefore as follows: The first component $\tilde{X}$ is indeed a piece-wise deterministic process, switching between states 0 and 1. The switching rate at time *t* for jumps from 0 to 1 is just given by the value of the second component $\tilde{Y}(t)$, and from 1 to 0 with complementary rate $1-\tilde{Y}(t)$. In-between jump times of $\tilde{X}$, the second component $\tilde{Y}$ behaves deterministically, following the equation

$$\begin{aligned} \mathrm{d} \tilde{Y}(t) = K(\tilde{X}(t) -\tilde{Y}(t))\mathrm{d}t, \end{aligned}$$

So while $\tilde{X}(t)$ is in state 0, $\tilde{Y}(t)$ decreases deterministically with exponential rate $-K\tilde{Y}(t)$, and while $\tilde{X}(t)$ is in state 1, $\tilde{Y}(t)$ increases with exponential rate $K(1-\tilde{Y}(t))$. This is illustrated in Fig. 2c.

*Interpretation: dormancy versus genetic drift on different timescales* In the classical Wright-Fisher model without dormancy, genetic drift drives the frequency process $(Z(t))_{t\ge 0}$ of a given allele towards the boundaries 0 and 1, where it fixates. This occurs on timescales of the order the (effective total) population size.

In the weak seed bank regime frequencies are described by $(\tilde{Z}(t))_{t\ge 0}$ and genetic drift is ‘slowed down’ in a quantitative sense by a factor $\beta ^2$, since dormant individuals may jump generations, increasing the effective population size accordingly. For example, expected fixation times will be stretched by the factor $\beta ^{-2}$.

In the strong seed bank regime, dormancy times and genetic drift both act on the same timescale. The resulting additional seed bank ‘island’ in the diffusion $(X(t),Y(t))_{t \ge 0}$ will slow down the effect of genetic drift in a *qualitative* sense. In fact, although the active population may fixate briefly in 0 or 1, the seed bank component will then quickly reintroduce variability via the migration term, hence the memory in the seed bank prevents final fixation in finite time (at least for non-trivial initial states). This interesting effect is discussed in detail in Blath et al. (2019), where it is also shown that the seed bank introduces ‘variability’ into the population model in a suitable sense, by means of a delay-equation reformulation of the seed bank diffusion.

Finally, in the extreme case where dormancy periods are much longer than the timescale of genetic drift, if time is measured in the super-evolutionary scale, fixation/extinction in the active population of $(\tilde{X}(t), \tilde{Y}(t))_{t\ge 0}$ will happen *instantaneously*, and last for a finite time. The switches of the frequency in the active population between 0 and 1 can be explained as follows: When a single ‘ancient’ allele ‘resuscitates’, it will usually not be able to fixate in the population and go extinct again. However, on the super-evolutionary timescale, these ‘trials’ reoccur many times, and eventually a resuscitating allele will fixate. If it is of the same type as the allele currently present in the active population, nothing changes and there will be no jump. However, if it is of the other type, this will cause $\tilde{X}$ to switch to the opposite boundary. The probabilities of the allele resuscitating at time *t* being of the given type or of the opposite type are $\tilde{Y}(t)$ and $1-\tilde{Y}(t)$, which explains the form of the rates in Theorem 6.

These observations regarding fixation or coexistence of types can be summed up as follows. In the Wright-Fisher diffusion without mutation $(Z(t))_{t\ge 0}$, ultimately, one type will fixate. In the weak seed bank regime described by $(\tilde{Z}(t))_{t \ge 0}$, there will also be one type that fixates, but the (expected) time until this happens is increased by a factor of $\beta ^{-2}$. In the strong seed bank regime, we will occasionally see fixation of one type in the active population, but then the seed bank will reintroduce variability immediately, so that coexistence is visible almost all the time. Finally, in the case of dormancy on the super-evolutionary timescale, at any given time, the active population will always be homomorphic, but the dominant type will switch from time to time, and there are no visible periods of coexistence at all.

*Duality and genealogical interpretation of the scaling regimes*

As we have seen, the processes describing the forward-in-time frequency of a given allele in a Wright-Fisher model with seed bank have natural dual processes describing their genealogies. Such genealogical processes shed light on the effect of dormancy on the ancestral processes of samples, but are also useful tools for the proofs of the previous theorems, as they tend to be mathematically simpler objects. Our new scaling regime is no exception.

**Fig. 3:**
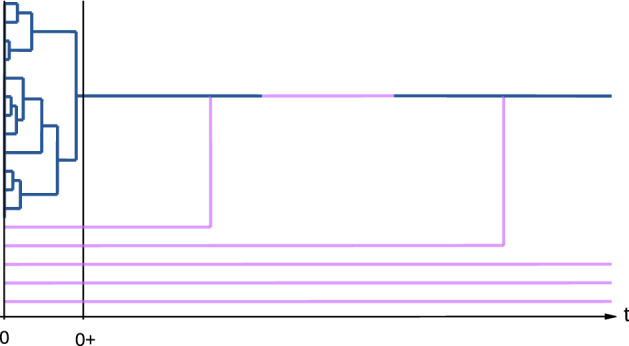
A typical realisation of an ancient ancestral lines process. Blue lines are active lineages, purple lines are dormant. At the macroscopic time-scale coalescence occurs instantaneously, which is what we see between the times 0 and $0+$. Afterwards we have at most one active lineage at any given time. If a dormant lineage activates, it coalesces immediately with the active lineage (colour figure online)

In the super-evolutionary scaling regime of Theorem 1.3 we obtain the block-counting process of the *ancient ancestral lines process* as a scaling limit of the genealogies (see Theorem 1.5 below). Intuitively, since we are considering a population for which dormancy times are of a larger order than the times of coalescences, at the super-evolutionary timescale, coalescences occur instantaneously, while migration between the active and the dormant state occurs at order 1, cf. Fig. 3. Hence, in the limit, for each time $t > 0$, there will be at most one active line. More formally, we obtain the following definition.

### Definition 1.4

(*The ancient ancestral lines process*) Let $(n_0,m_0) \in \mathbb {N}_0 \times \mathbb {N}_0$. The $(n_0,m_0)$-*ancient ancestral lines process* is the continuous-time Markov chain $(\tilde{N}(t), \tilde{M}(t))_{t \ge 0}$ with initial value $(\tilde{N}(0), \tilde{M}(0))=(n_0, m_0)$, taking values in the state space

$$\begin{aligned} E_{(n_0,m_0)}:=\lbrace 0, \dots , n_0+m_0 \rbrace ^2, \end{aligned}$$

with semi-group

$$\begin{aligned} \Pi (t):=Pe^{tG}, \qquad t>0, \end{aligned}$$

where $\Pi (0)$ is defined as $\mathrm{I}_E$, the identity on $E_{(n_0,m_0)}$. *P* is a projection ($P^2=P$) given by

7 $$\begin{aligned} P_{(n,m),({\bar{n}},{\bar{m}})} := {\left\{ \begin{array}{ll} 1, &{} \text { if } {\bar{n}} = 1,\, n \ge 1,\, {\bar{m}} = m,\\ 1, &{} \text { if } {\bar{n}} = n = 0,\, {\bar{m}} = m,\\ 0, &{} \text { otherwise,} \end{array}\right. } \end{aligned}$$

for all $(n,m),\,({\bar{n}}, {\bar{m}}) \in E_{(n_0,m_0)}$ and *G* is defined as

$$\begin{aligned} G_{(n,m),({\bar{n}},{\bar{m}})} := {\left\{ \begin{array}{ll} Km, &{} \text { if } {\bar{n}} = 1,\, n \ge 0,\, {\bar{m}} = m-1,\\ 1, &{} \text { if } {\bar{n}} = 0,\, n \ge 1,\, {\bar{m}} = m+1,\\ -1-Km, &{} \text { if } {\bar{n}} = 1,\, n \ge 1,\, {\bar{m}} = m,\\ -Km, &{} \text { if } {\bar{n}} = n = 0,\, {\bar{m}} = m,\\ 0, &{} \text { otherwise.} \end{array}\right. } \end{aligned}$$

Note the form of the semi-group of the Markov chain which in particular is not standard, i.e. $\lim _{t\downarrow 0} \Pi (t) = P \ne \mathrm {Id}_E$ (cf. Chung 1960). Since the projection *P* acts for all $t>0$, this process takes values in the smaller space $\{0,1\}\times \{0, \ldots , m_0+1\}$ $\mathbb {P}$-a.s. for every (fixed) $t>0$. The first two “rates” given in the definition of *G* correspond to the events of resuscitation (with immediate coalescence if applicable) and initiation of dormancy. *G* is, however, not a *Q*-matrix, since for any ${\bar{n}} \ge 2$ it has negative values off the diagonal. These only regard states that will be collapsed by *P* into the smaller state space.

The technical challenges due to the degenerate form of the semi-group of the scaling limit coming from “separation of timescales phenomena” (cf. for example Wakeley 2009, Chapter 6 from the population genetics perspective) require special care as we detail in Sect. 2.1. Subsequently, we apply the above strategy to our model in Sect. 2.2 proving that the *ancient ancestral lines process* arises as the scaling limit of the block-counting process of the seed bank coalescent in the sense of convergence of the finite-dimensional distributions.

### Theorem 1.5

Denote by $(N^c(t), M^c(t))_{t \ge 0}$ the block counting process of the seed bank coalescent as defined in Definition 1.2 with migration rate $c>0$ and assume that it starts at some $(n_0, m_0) \in \mathbb {N}\times \mathbb {N}$, $\mathbb {P}$-a.s.

Furthermore let $(\tilde{N}(t), \tilde{M}(t)))_{t \ge 0}$ be the ancient ancestral lines process from Definition 1.4 with the same initial condition. Then, for any sequence of migration rates $(c_{\kappa })_{\kappa \in \mathbb {N}}$ with $c_{\kappa } \rightarrow 0$ when $\kappa \rightarrow \infty$, we have

$$\begin{aligned} \left( N^{c_{\kappa }}\left( \frac{1}{c_{\kappa }} t\right) , M^{c_{\kappa }}\left( \frac{1}{c_{\kappa }} t\right) \right) _{t \ge 0} \xrightarrow {\mathrm{f.d.d.}} \big (\tilde{N}(t), \tilde{M}(t)\big )_{t \ge 0}. \end{aligned}$$

Without loss of generality, we assume $(\tilde{N}(t), \tilde{M}(t))_{t \ge 0}$ to be càdlàg.

*Spontaneous and simultaneous switching*

One should note that for the above models, we assumed a ‘spontaneous’ switching. ‘Simultaneous’ switching, where transition to and from the dormant population are triggered by environmental cues, are currently an active area of research, see e.g. Blath et al. (2020a).

## Scaling limits for continuous-time Markov chains

Motivated by the example of the *super-evolutionary scaling* in the introductory section, as a first step, we consider scaling limits of continuous-time Markov chains. Indeed, when speeding up time, some transition rates diverge to $\infty$, thus obstructing direct *Q*-matrix computations and producing states that are vacated immediately. This effect is frequently observed when dealing with “separation of timescales phenomena” and can in a ‘well-behaved’ scenario still lead to a scaling limit with potentially “degenerate”, i.e. non-standard transition semi-group of the form

$$\begin{aligned} Pe^{tG}, \quad t \ge 0, \end{aligned}$$

where *P* is a *projection* to a subspace of the original state space as a result of “immediately vacated states” and satisfies $G=PG=GP$. For *discrete-time* Markov chains, this situation was considered e.g. in Möhle (1998), Birkner et al. (2013) and recently also Möhle and Notohara (2016). Since the handling of such situations for *continuous-time* Markov chains (such as the above block counting process) might be of general interest and is somewhat more involved than the discrete case, we give a detailed “recipe” for such convergence proofs in Sect. 2.1. Note that all of these results can in principle be seen as specialised and ready-to-use variants of the general operator-theoretic scheme derived in Kurtz (1973) in the context of ‘random evolutions’ (see also Ethier and Kurtz 1986, Sect. 1.7). Recent applications of this scheme can also be found in Bobrowski (2015).

### Separation of timescales phenomena for continuous-time Markov chains: a strategy

Given a sequence of continuous-time Markov chains $(\xi ^{\kappa }(t))_{t\ge 0}$, $\kappa \in \mathbb {N}$ with finite state-space *E* (equipped with a metric *d*), suppose that our aim is to prove its convergence in finite-dimensional distributions under a suitable time-rescaling $(C_{\kappa })_{\kappa \in \mathbb {N}}$ to a continuous-time Markov chain $(\xi (t))_{t\ge 0}$ when $\kappa \rightarrow \infty$.

Our programme to carry out such a proof has two steps:

First, consider an appropriate *time discretisation* of $(\xi ^{\kappa }(t))_{t\ge 0}$, $\kappa \in \mathbb {N}$. Employing the machinery from Birkner et al. (2013), Möhle (1998) and Möhle and Notohara (2016) available in this context, one can prove convergence of a rescaling of the discretised processes to a continuous-time Markov chain $(\xi (t))_{t\ge 0}$ when $\kappa \rightarrow \infty$ in the sense of weak convergence in finite-dimensional distributions.

Second, we prove a *continuity result* to show that the suitably rescaled original process converges in finite-dimensional distributions to the same limit.

In order to formulate the conditions on the time-rescaling and the original sequence of Markov chains, we rewrite the time-rescaling as $C_{\kappa }=b_{\kappa }/a_{\kappa }$, where further assumptions on the non-negative sequences $(a_{\kappa })_{\kappa \in \mathbb {N}}$ and $(b_{\kappa })_{\kappa \in \mathbb {N}}$ will be specified below.

**Step (i) Time discretisation and its convergence**

The following lemma is an immediate application of Lemma 1.7 in Birkner et al. (2013) analogous to Theorem 1 in Möhle (1998). We rephrase it in this framework for the convenience of the reader and as reference for the examples we will consider below.

Observe that for a non-negative sequence $(a_{\kappa })_{\kappa \in \mathbb {N}}$, $(\xi ^{\kappa }(i/a_{\kappa }))_{i \in \mathbb {N}}$ is a discrete-time Markov chain with finite state-space *E* for each $\kappa \in \mathbb {N}$. We equip the matrices $A = (A_{e,{\bar{e}}})_{e, {\bar{e}} \in E}$ on *E* with the matrix norm $\Vert A \Vert :=\max _{e \in E}\sum _{{\bar{e}} \in E} A_{e,{\bar{e}}}$. Since *E* is finite, convergence in the matrix norm is equivalent to pointwise convergence.

#### Lemma 2.1

Let $(a_{\kappa })_{\kappa \in \mathbb {N}}$ and $(b_{\kappa })_{\kappa \in \mathbb {N}}$ be non-negative sequences such that $a_{\kappa }$, $b_{\kappa }$, $b_{\kappa }/a_{\kappa } \rightarrow \infty$ as $\kappa \rightarrow \infty$. For each $\kappa \in \mathbb {N}$ denote by $\Pi _{\kappa }$ the transition matrix of the discrete-time, time-homogeneous Markov chain $(\xi ^{\kappa }(i/a_{\kappa }))_{i \in \mathbb {N}}$.

Assume that for every $\kappa \in \mathbb {N}$ we have a representation of the transition matrix of the form

8 $$\begin{aligned} \Pi _\kappa = A_\kappa + \frac{1}{b_{\kappa }}B_\kappa , \end{aligned}$$

such that the following holds: $A_{\kappa }$ is a stochastic matrix and

9 $$\begin{aligned} \lim _{C \rightarrow \infty }\lim _{\kappa \rightarrow \infty } \sup _{r \ge Ca_{\kappa }} \Vert ({{A}_{\kappa }})^r -P\Vert =0 \end{aligned}$$

for some matrix *P*. Furthermore, we require that the matrix limit with respect to the matrix norm

10 $$\begin{aligned} G:=\lim _{\kappa \rightarrow \infty } P {B}_{\kappa } P \qquad \text {exists}. \end{aligned}$$

Then, we obtain the following convergence (with respect to the matrix norm):

11 $$\begin{aligned} \lim _{\kappa \rightarrow \infty }\Pi _\kappa ^{\lfloor t b_{\kappa }\rfloor } = \lim _{\kappa \rightarrow \infty } \left( {A}_{\kappa } + \frac{1}{b_{\kappa }}{B}_{\kappa }\right) ^{\lfloor t b_{\kappa }\rfloor } = Pe^{tG} =:\Pi (t) \qquad \text {for all}\;\; t > 0. \end{aligned}$$

In particular, if we define $\Pi (0):=\text {Id}_E$, then $(\Pi (t))_{t\ge 0}$ is a semi-group that generates a continuous-time Markov chain which we denote by $(\xi (t))_{t\ge 0}$.

If $\xi ^{\kappa }(0) \xrightarrow {w} \xi (0)$ as $\kappa \rightarrow \infty$, Eq. (11) implies

$$\begin{aligned} \left( \xi ^\kappa \left( \frac{\lfloor b_{\kappa }t\rfloor }{a_{\kappa }}\right) \right) _{t\ge 0} \xrightarrow {\mathrm{f.d.d.}} (\xi (t))_{t\ge 0}, \quad \text { as } \kappa \rightarrow \infty . \end{aligned}$$

Here, $\xrightarrow {w}$ denotes weak convergence.

Before proceeding to the proof of this lemma, let us make a few remarks about the assumptions and results observed in it.

#### Remark 2.2

1. Since $\Pi _{\kappa }$ is the transition matrix of the $(\xi ^{\kappa }(t))_{t\ge 0}$ under a time-change by $a_{\kappa }^{-1}$, in a representation like (8), $A_\kappa$ is a stochastic matrix that contains only entries of order 1 and $a_\kappa ^{-1}$, and $B_\kappa$ contains only entries of order 1 and *o*(1). Since we then speed-up time by a factor $b_{\kappa }$, we obtain a separation of timescales, where the entries in $A_{\kappa }$ give rise to a projection matrix *P* acting on the probability distributions on *E*, while the entries in $B_{\kappa }$ give rise to a “Q-matrix”. The $A_{\kappa }$ contain the transition rates of $(\xi ^{\kappa }(t))_{t\ge 0}$ that occur at a faster rate than the new timescale, hence they occur “instantaneously” in the limit. The entries in $B_{\kappa }$ correspond to the transitions of $(\xi ^{\kappa }(t))_{t\ge 0}$ that either occur on the new timescale or are slower, hence describing the transitions visible in the limit and those that vanish.
2. Note that given (9), the matrix *P* is necessarily a projection on *E*, i.e. satisfies $P^2=P$. Since $P=P^2$, we have $PG=GP=G$ and hence $Pe^{tG}=e^{tG}P = P-I+e^{tG}$ for any $t\ge 0$. In particular, $(\Pi (t))_{t\ge 0}$ is not standard, as $\lim _{t\downarrow 0}\Pi (t) = P \ne \Pi (0) = \mathrm {Id}_E$. *P* effectively restricts the state-space of the limiting chain to a subspace of *E*.
  Observe that *G* differs from a normal *Q*-matrix as it may have negative entries off the diagonal.

#### Proof of Lemma 2.1

Conditions (8), (9) and (10) above are precisely conditions (36), (46) and (48) in Birkner et al. (2013). Hence (11) is the claim of (49) in Lemma 1.7 and Remark 1.8 in Birkner et al. (2013). Remark 2.2 in particular implies that the Chapman-Kolmogorov equations hold for $(\Pi (t))_{t\ge 0}$ and hence this generates a continuous-time Markov chain which we denote by $(\xi (t))_{t\ge 0}$ (see, for example, Kallenberg 2002, Thm. 8.4). The convergence in Eq. (11) and the Markov property then imply the convergence in finite-dimensional distributions.$\square$

**Step (ii) Convergence of the continuous-time Markov chains**

The previous step ensured the existence of a limit for suitably discretised versions of the original sequence of continuous-time Markov chains $(\xi ^{\kappa }(t))_{t\ge 0}$. The following lemma tells us under what conditions such a discretisation is sufficiently fine to also imply the convergence of the $(\xi ^{\kappa }(t))_{t\ge 0}$ to the same limit.

#### Lemma 2.3

Let $(\xi ^\kappa (t))_{t \ge 0},\kappa \in \mathbb {N}$ be a sequence of continuous-time, time-homogeneous Markov chains with finite state space *E* (equipped with some metric *d*). Let $(a_{\kappa })_{\kappa \in \mathbb {N}}$ and $(b_{\kappa })_{\kappa \in \mathbb {N}}$ be non-negative sequences.

Denote by $G^{\kappa }$ the *Q*-matrix of $(\xi ^\kappa (t))_{t \ge 0}$ for each $\kappa \in \mathbb {N}$ and set $q_{\kappa }:=\max _{e \in E} \left\{ -G^{\kappa }_{e,e}\right\}$. If

- $$\frac{q_{\kappa }}{a_{\kappa }} \rightarrow 0$$ as $$\kappa \rightarrow \infty $$, and
- $$\left( \xi ^\kappa \left( \frac{\lfloor b_{\kappa }t\rfloor }{a_{\kappa }}\right) \right) _{t \ge 0} \xrightarrow {\mathrm{f.d.d.}} (\xi (t))_{t \ge 0}$$ as $$\kappa \rightarrow \infty $$,

then also

$$\begin{aligned} \left( \xi ^\kappa \left( \frac{b_{\kappa }}{a_{\kappa }}t\right) \right) _{t \ge 0} \xrightarrow {\mathrm{f.d.d.}} (\xi (t))_{t \ge 0} \quad \text { as }\kappa \rightarrow \infty . \end{aligned}$$

#### Proof

When started at $e \in E$, the time to the first jump of $\xi ^{\kappa }$ is exponentially distributed with parameter $-G^{\kappa }_{e,e}$. Hence on sees that condition a) was chosen precisely such that

12 $$\begin{aligned} \mathbb {P}\left\{ (\xi ^{\kappa }(t))_{t\ge 0} \text { has a jump in } \left( 0\;,\;\frac{1}{a_{\kappa }}\right] \right\} \le 1-\exp \left( \frac{-q_{\kappa }}{a_{\kappa }}\right) \rightarrow 0, \quad \kappa \rightarrow \infty . \end{aligned}$$

Observe that for the distance between $\xi ^{\kappa }\left( \frac{b_{\kappa }t}{a_{\kappa }}\right)$ and $\xi ^{\kappa }\left( \frac{\lfloor b_{\kappa }t\rfloor }{a_{\kappa }}\right)$ at any time $t \ge 0$ we have

$$\begin{aligned} d\left( \xi ^{\kappa }\left( \frac{b_{\kappa }t}{a_{\kappa }}\right) , \xi ^{\kappa }\left( \frac{\lfloor b_{\kappa }t\rfloor }{a_{\kappa }}\right) \right) > 0 \end{aligned}$$

*only if* the process $(\xi ^{\kappa }(t))_{t\ge 0}$ has a jump in the interval $\left( \frac{\lfloor b_{\kappa }t\rfloor }{a_{\kappa }}, \frac{b_{\kappa }t}{a_{\kappa }}\right]$. Since the length of this interval can be estimated through

$$\begin{aligned} 0 \le \frac{b_{\kappa }t}{a_{\kappa }} - \frac{\lfloor b_{\kappa }t\rfloor }{a_{\kappa }} \le \frac{1}{a_{\kappa }} \end{aligned}$$

and the Markov chains are time-homogeneous we can in turn estimate the probability of a jump in the interval using (12) and obtain

13 $$\begin{aligned} \mathbb {P}\left\{ d\left( \xi ^{\kappa }\left( \frac{b_{\kappa }t}{a_{\kappa }}\right) , \xi ^{\kappa }\left( \frac{\lfloor b_{\kappa }t\rfloor }{a_{\kappa }}\right) \right) >0\right\}&\le \mathbb {P}\left\{ (\xi ^{\kappa }(t))_{t\ge 0} \text { has a jump in } \left( \frac{\lfloor b_{\kappa }t\rfloor }{a_{\kappa }}, \frac{b_{\kappa }t}{a_{\kappa }}\right] \right\} \nonumber \\&\le 1-\exp \left( \frac{-q_{\kappa }}{a_{\kappa }}\right) \rightarrow 0, \quad \kappa \rightarrow \infty . \end{aligned}$$

In order to prove the convergence of the finite-dimensional distributions, recall that weak convergence of measures is equivalent to convergence in the Prohorov metric (see, e.g. Whitt (2002), Section 3.2). Hence, assumption (b) yields that for all time points $0 \le t_0, \ldots , t_l<\infty$, states $e_0, \ldots , e_l \in E$ and any $\varepsilon >0$ sufficiently small there exists a $\bar{\kappa } \in \mathbb {N}$ such that for all $\kappa \ge \bar{\kappa }$:

$$\begin{aligned} \mathbb {P}\left\{ \xi ^{\kappa }\left( \frac{\lfloor b_{\kappa }t_0\rfloor }{a_{\kappa }}\right) =e_0, \ldots , \xi ^{\kappa }\left( \frac{\lfloor b_{\kappa }t_l\rfloor }{a_{\kappa }}\right) =e_l\right\} \ge \mathbb {P}\left\{ \xi (t_0)=e_0, \ldots , \xi (t_l)=e_l\right\} - \frac{\varepsilon }{2}. \end{aligned}$$

Combining this with (13) we see that for all time points $0 \le t_0, \ldots , t_l<\infty$, states $e_0, \ldots , e_l \in E$ and any $\varepsilon >0$ sufficiently small there exists a $\bar{\kappa } \in \mathbb {N}$ such that for all $\kappa \ge \bar{\kappa }$

$$\begin{aligned} \mathbb {P}&\left\{ \xi ^{\kappa }\left( \frac{ b_{\kappa }t_0}{a_{\kappa }}\right) =e_0, \ldots , \xi ^{\kappa }\left( \frac{b_{\kappa }t_l}{a_{\kappa }}\right) =e_l\right\} \\&\qquad \ge \mathbb {P}\Bigg \{ \xi ^{\kappa }\left( \frac{\lfloor b_{\kappa }t_0\rfloor }{a_{\kappa }}\right) =e_0, \ldots , \xi ^{\kappa }\left( \frac{\lfloor b_{\kappa }t_l\rfloor }{a_{\kappa }}\right) =e_l, \\&\qquad \qquad \qquad d\left( \xi ^{\kappa }\left( \frac{ b_{\kappa }t_0}{a_{\kappa }}\right) , \xi ^{\kappa }\left( \frac{\lfloor b_{\kappa }t_0\rfloor }{a_{\kappa }}\right) \right) =\cdots = d\left( \xi ^{\kappa }\left( \frac{ b_{\kappa }t_l}{a_{\kappa }}\right) , \xi ^{\kappa }\left( \frac{\lfloor b_{\kappa }t_l\rfloor }{a_{\kappa }}\right) \right) = 0\Bigg \}\\&\qquad \ge \mathbb {P}\left\{ \xi ^{\kappa }\left( \frac{\lfloor b_{\kappa }t_0\rfloor }{a_{\kappa }}\right) =e_0, \ldots , \xi ^{\kappa }\left( \frac{\lfloor b_{\kappa }t_l\rfloor }{a_{\kappa }}\right) =e_l \right\} - \frac{\varepsilon }{2}\\&\qquad \ge \mathbb {P}\left\{ \xi (t_0)=e_0, \ldots , \xi (t_l)=e_l\right\} - \varepsilon . \end{aligned}$$

This implies the convergence of the finite-dimensional distributions of $\left( \xi ^{\kappa }\left( \frac{b_{\kappa }}{a_{\kappa }}t\right) \right) _{t \ge 0}$ to the finite-dimensional distributions of $(\xi (t))_{t \ge 0}$ in the Prohorov metric and hence weakly, which completes the proof. $\square$

### The ancient ancestral lines process (and other scaling limits)

Let us apply this machinery to the *“ancestral lines process”* introduced in Sect. 1. Indeed, consider the block-counting process of the seed bank coalescent defined in Definition 1.2 with vanishing migration rate *c*.

If we let $c\rightarrow 0$ and simultaneously speed up time by a factor $1/c \rightarrow \infty$, we obtain a new structure given in Definition 1.4, thus uncovering a separation-of-timescales phenomenon. Theorem 1.5 formalises this heuristic and establishes the ancient ancestral lines process as scaling limit in finite-dimensional distributions of the block-counting process of the seed bank coalescent. Note that indeed *P* is a projection matrix and $PG=GP=G$, for *P* and *G* as in Definition 1.4.

#### Proof of Theorem 1.5

Let $(c_{\kappa })_{\kappa \in \mathbb {N}}$ be a positive sequence such that $c_{\kappa }\rightarrow 0$. Without loss of generality assume $c_{\kappa } \le 1$ for all $\kappa \in \mathbb {N}$. We prove the result using the machinery outlined in the previous section with $a_{\kappa } := c^{-2}_{\kappa }$ and $b_{\kappa } := c^{-3}_{\kappa }$.

Recall that $(N^{c_{\kappa }}(t), M^{c_{\kappa }}(t))_{t \ge 0}$ is the block counting process of the seed bank coalescent as defined in Definition 1.2 with migration rate $c_{\kappa }>0$ and assume that it starts at some $(n_0, m_0) \in \mathbb {N}\times \mathbb {N}$, $\mathbb {P}$-a.s. Let $\mathbb {N}_0$ be equipped with the discrete topology.

**Step (i)** In analogy to the notation in the previous section we abbreviate

$$\begin{aligned} (\xi ^{\kappa }(t))_{t \ge 0}:=(N^{c_{\kappa }}(t), M^{c_{\kappa }}(t))_{t \ge 0} \end{aligned}$$

and consider a discretised process with time steps of length $a_{\kappa }^{-1} = c^{2}_{\kappa }$ by defining

$$\begin{aligned} \eta ^{\kappa }(i):= \xi ^{\kappa }(ic^2_{\kappa }), \qquad i \in \mathbb {N}_0. \end{aligned}$$

Let $\Pi _{\kappa }$ be the transition matrix of the Markov chain $(\eta ^\kappa (i))_{i \in \mathbb {N}_0}$. The transition probabilities of this chain are

14 $$\begin{aligned} (\Pi _{\kappa })&_{(n,m),({\bar{n}}, {\bar{m}})} = \mathbb {P}\{\eta ^\kappa (1) = ({\bar{n}},{\bar{m}}) \mid \eta ^\kappa (0) = (n,m)\} \nonumber \\&= \mathbb {P}\left\{ (N^{c_{\kappa }}(c^2_{\kappa }), M^{c_{\kappa }}(c^2_{\kappa })) = ({\bar{n}},{\bar{m}}) \mid (N^{c_{\kappa }}(0), M^{c_{\kappa }}(0)) = (n,m)\right\} \nonumber \\&= {\left\{ \begin{array}{ll} \left( {\begin{array}{c}n\\ 2\end{array}}\right) c^2_{\kappa } + o(c^3_{\kappa }), &{} \text { if } {\bar{n}} = n - 1,\, {\bar{m}} = m,\\ c_{\kappa }nc^2_{\kappa } + o(c^3_{\kappa }), &{} \text { if } {\bar{n}} = n - 1,\, {\bar{m}} = m+1,\\ c_{\kappa }Kmc^2_{\kappa } + o(c^3_{\kappa }), &{} \text { if } {\bar{n}} = n+1,\, {\bar{m}} = m-1,\\ 1 - \left( {\begin{array}{c}n\\ 2\end{array}}\right) c^2_{\kappa } - o(c^2_{\kappa }) - c_{\kappa }nc^2_{\kappa } - c_{\kappa }Kmc^2_{\kappa } - o(c^3_{\kappa }), &{} \text { if } {\bar{n}} = n,\, {\bar{m}} = m,\\ o(c^3_{\kappa }), &{} \text { otherwise,} \end{array}\right. } \end{aligned}$$

for any sensible $(n,m), \, ({\bar{n}}, {\bar{m}}) \in E_{(n_0,m_0)}$, recalling the convention of $$\left( {\begin{array}{c}n\\ 2\end{array}}\right) =0$$ for $n\le 1$. This can be seen as follows.

Denote by $T_1$ the time of the first jump of $\xi ^{\kappa }$ and by $T_2$ the time between the first and the second jump of $\xi ^{\kappa }$. By the strong Markov property we know that $T_1$ and $\xi ^{\kappa }(T_1)$, as well as $T_1$ and $T_2$ are independent. Conditioning on $\xi ^{\kappa }$ to start in (*n*, *m*), we also know that $T_1$ follows an exponential distribution with parameter $$\left( {\begin{array}{c}n\\ 2\end{array}}\right) + c_{\kappa }n + c_{\kappa }Km$$ and that $T_2$ dominates an exponential random variable with parameter $$2\left( {\begin{array}{c}n-1\\ 2\end{array}}\right) + \left( {\begin{array}{c}n+1\\ 2\end{array}}\right) + c_{\kappa }(3n+1+ 3Km)$$ (condition on the possible values of $\xi ^{\kappa }(T_1)$, then take the minimum of the possible exponential random variables describing the waiting time to the next jump). Using this one can check that

15 $$\begin{aligned} \mathbb {P}&\left\{ T_1+T_2 \le c_{\kappa }^2\right\} \in o(c_{\kappa }^3). \end{aligned}$$

To calculate the transition probabilities in (14), note that (15) tells us that the probability of seeing more than one jump by $\xi ^{\kappa }$ in the interval $[0,c_{\kappa }^2]$ is in $o(c_{\kappa }^3)$. In particular, this gives us the order of the transition probabilities for $\eta ^{\kappa }$ to states summarised under “otherwise”, i.e. those that require more than one jump by $\xi ^{\kappa }$. The transitions that are possible with just one jump are “coalescence”, “dormancy” and “resuscitation” in the order in which they appear in (14). We calculate the case of “coalescence”: Note that in order to see such a transition at least one jump must have happened. Hence,

$$\begin{aligned} \mathbb {P}\{\eta ^\kappa (1)&= (n-1, m) \mid \eta ^\kappa (0) = (n,m)\} \\&= \mathbb {P}\{\xi ^\kappa (T_1) = (n-1, m), T_1 \le c_{\kappa }^2, \; T_1+T_2 > c_{\kappa }^2 \mid \eta ^\kappa (0) = (n,m)\} + o(c_{\kappa }^3) \\&= \mathbb {P}\{\xi ^\kappa (T_1) = (n-1, m), T_1 \le c_{\kappa }^2 \mid \eta ^\kappa (0) = (n,m)\} + o(c_{\kappa }^3) \\&= \frac{\left( {\begin{array}{c}n\\ 2\end{array}}\right) }{\left( {\begin{array}{c}n\\ 2\end{array}}\right) + c_{\kappa }(n+Km)} \left( 1 - \mathbb {P}\left\{ T_1 \ge c_{\kappa }^2\right\} \right) + o(c_{\kappa }^3) = \left( {\begin{array}{c}n\\ 2\end{array}}\right) c_{\kappa }^2 + o(c_{\kappa }^3) \end{aligned}$$

where we used (15) for the third equality, the independence of $\xi ^{\kappa }(T_1)$ and $T_1$ for the fourth and a Taylor expansion and the distribution of $T_1$ for the fifth equality. The transition probabilities for “dormancy” and “resuscitation” can be calculated analogously. The calculation of the transition probability to the same state the chain originated from is obvious.

With the representation in (14) we now obtain the decomposition as in (8)

$$\begin{aligned} \Pi _{\kappa } = A_{\kappa } + \frac{B_\kappa }{b_{\kappa }} \end{aligned}$$

with $b_{\kappa } = c^{-3}_{\kappa }$ as defined above and

$$\begin{aligned} {(A_{\kappa })}_{(n,m),({\bar{n}}, {\bar{m}})} = {\left\{ \begin{array}{ll} \left( {\begin{array}{c}n\\ 2\end{array}}\right) c^2_{\kappa }, &{} \text { if } {\bar{n}} = n - 1,\, {\bar{m}} = m,\\ 1 - \left( {\begin{array}{c}n\\ 2\end{array}}\right) c^2_{\kappa }, &{} \text { if } {\bar{n}} = n,\, {\bar{m}} = m,\\ 0, &{} \text { otherwise,} \end{array}\right. } \end{aligned}$$

and

16 $$\begin{aligned} {(B_{\kappa })}_{(n,m),({\bar{n}}, {\bar{m}})} = {\left\{ \begin{array}{ll} n + o(1), &{} \text { if } {\bar{n}} = n - 1,\, {\bar{m}} = m+1,\\ Km + o(1), &{} \text { if } {\bar{n}} = n+1,\, {\bar{m}} = m-1,\\ - n - Km + o(1), &{} \text { if } {\bar{n}} = n,\, {\bar{m}} = m,\\ o(1), &{} \text { otherwise.} \end{array}\right. } \end{aligned}$$

In order to apply Lemma 2.1, we now need to check condition (9), i.e.

17 $$\begin{aligned} \lim _{C \rightarrow \infty }\lim _{\kappa \rightarrow \infty } \sup _{r \ge Cc^{-2}_{\kappa }} \Vert ({A}_{\kappa })^r - P\Vert = 0 \end{aligned}$$

for *P* given in (7). Since $A_{\kappa }$ is a stochastic matrix, let $(Z^{\kappa }_r)_{r \in \mathbb {N}_0}$ be the Markov chain associated to it. This is a pure death process in the first component and constant in the second. By definition of the matrix norm, we get

$$\begin{aligned} \Vert (A_{\kappa })^r -P \Vert&= \max _{(n,m) \in E_{(n_0, m_0)}} \sum _{({\bar{n}}, {\bar{m}}) \in E_{(n_0, m_0)}} |(A_{\kappa })^r_{(n,m), ({\bar{n}}, {\bar{m}})} - P_{(n,m), ({\bar{n}}, {\bar{m}})}| \\&= \max _{(n,m) \in E_{(n_0, m_0)}} \Big ( \vert (A_{\kappa })^r_{(n,m), (1,m)} - 1 \vert + \sum _{{\bar{n}} = 2}^{n} \vert (A_{\kappa })^r_{(n,m), ({\bar{n}},m)} - 0 \vert \Big ) \\&= \max _{(n,m) \in E_{(n_0, m_0)}} 2\Big ( 1-(A_{\kappa })^r_{(n,m), (1,m)}\Big ) \\&= 2 \max _{(n,m) \in E_{(n_0, m_0)}} \mathbb {P}\big \{ Z^{\kappa }_r \ne (1,m) \mid Z^{\kappa }_0 = (n,m)\big \}\\&= 2 \mathbb {P}\big \{ Z^{\kappa }_r \ne (1,m_0) \mid Z^{\kappa }_0 = (n_0,m_0)\big \} \end{aligned}$$

Observe that for all $n \in \{ 2, \ldots , n_0\}$ (and all $m \in \{ 0, \ldots , m_0\}$) the probability of $Z^{\kappa }$ to jump to $(n-1, m)$ in the next step can be bounded:

$$\begin{aligned} (A_{\kappa })_{(n,m), (n-1,m)} = \left( {\begin{array}{c}n\\ 2\end{array}}\right) c^{2}_{\kappa }\ge c^{2}_{\kappa }. \end{aligned}$$

Hence, the number of time-steps required for $Z^{\kappa }$ to reach $(1,m_0)$ if it is started in $(n_0,m_0)$ is dominated by the sum of $n_0-1$ independent geometric random variables $\gamma _1^{\kappa },\dots ,\gamma _{n_0-1}^{\kappa }$ with success probability $c^{2}_{\kappa }$. More precisely, if we define $T:=\inf \{{\bar{r}} \in \mathbb {N}_0 \mid Z^{\kappa }_{{\bar{r}}} = (1, m_0)\}$, then

$$\begin{aligned} \mathbb {P}\big \{ Z^{\kappa }_r \ne (1,m_0) \mid Z^{\kappa }_0 = (n_0,m_0)\big \}&\le \mathbb {P}\big \{T\ge r \mid Z^{\kappa }_0 = (n_0,m_0)\big \} \\&\le \mathbb {P}\big \{ \gamma _1^{\kappa }+\cdots +\gamma _{n_0-1}^{\kappa } \ge r\big \}. \end{aligned}$$

By Markov’s inequality, we get

$$\begin{aligned} \mathbb {P}\left\{ \gamma _1^{\kappa } + \dots + \gamma _{n_0-1}^{\kappa } \ge r \right\}&\le \frac{1}{r} \mathbb {E}\big [\gamma _1^{\kappa } + \dots + \gamma _{n_0-1}^{\kappa } \big ] = \frac{1}{r}\frac{(n_0-1)}{c^{2}_{\kappa }}. \end{aligned}$$

Combining these observations we obtain

$$\begin{aligned} \lim _{C \rightarrow \infty }\lim _{\kappa \rightarrow \infty } \sup _{r \ge Cc^{-2}_{\kappa }} \Vert ({A}_{\kappa })^r - P\Vert&\le \lim _{C \rightarrow \infty }\lim _{\kappa \rightarrow \infty } \sup _{r \ge Cc^{-2}_{\kappa }} 2 \mathbb {P}\big \{ \gamma _1^{\kappa }+\cdots +\gamma _{n_0-1}^{\kappa } \ge r\big \} \\&= \lim _{C \rightarrow \infty }\lim _{\kappa \rightarrow \infty } 2 \mathbb {P}\big \{ \gamma _1^{\kappa }+\cdots +\gamma _{n_0-1}^{\kappa } \ge Cc^{-2}_{\kappa }\big \} \\&= \lim _{C \rightarrow \infty }\lim _{\kappa \rightarrow \infty }\frac{c^{2}_{\kappa }}{C}\frac{(n_0-1)}{c^{2}_{\kappa }}=0 \end{aligned}$$

and (17) holds. We are now left to establish the matrix-norm limit (10) and show that coincides with the *G* given in Definition 1.4. Notice that $B_{\kappa }$ itself converges when $\kappa \rightarrow \infty$ uniformly and in the matrix norm (recalling that the state space $E_{(n_0,m_0)}$ is finite):

$$\begin{aligned} B \; := \;\lim _{\kappa \rightarrow \infty } B_{\kappa } \; = \; {\left\{ \begin{array}{ll} n, &{} \text { if } {\bar{n}} = n-1,\, {\bar{m}} = m+1,\\ Km, &{} \text { if } {\bar{n}} = n+1,\, {\bar{m}} = m-1,\\ -n-Km, &{} \text { if } {\bar{n}} = n,\, {\bar{m}} = m,\\ 0, &{} \text { otherwise.} \end{array}\right. } \end{aligned}$$

Simply multiplying the matrices on the left-hand-side we obtain $PBP=G$ and therefore

18 $$\begin{aligned} \lim _{\kappa \rightarrow \infty }PB_{\kappa }P = PBP = G \end{aligned}$$

and thus (10). Since we have proven the assumptions, Lemma 2.1 yields

$$\begin{aligned} \lim _{\kappa \rightarrow \infty }\Pi _\kappa ^{\lfloor t c^{-3}_{\kappa }\rfloor } = \lim _{\kappa \rightarrow \infty } \left( {A}_{\kappa } + c^{3}_{\kappa }{B}_{\kappa }\right) ^{\lfloor t c^{-3}_{\kappa }\rfloor } = Pe^{tG} =:\Pi (t) \qquad \text {for all}\;\; t > 0, \end{aligned}$$

and under the additional assumption that $\eta ^{\kappa }(0) =(N^{c_{\kappa }}(0), M^{c_{\kappa }}(0)) = (\tilde{N}(0), \tilde{M}(0))$, also

$$\begin{aligned} (\eta ^\kappa (\lfloor c^{-3}_{\kappa }t\rfloor ))_{t\ge 0} \xrightarrow {\mathrm{f.d.d.}} (\tilde{N}(t),\tilde{M}(t))_{t\ge 0}, \qquad \kappa \rightarrow \infty , \end{aligned}$$

where $(\tilde{N}(t),\tilde{M}(t))_{t\ge 0}$ is the ancient ancestral lines process defined in Definition 1.4.

**Step (ii)** We would now like to apply Lemma 2.3. Denote by $Q^{c_{\kappa }}$ the *Q*-matrix of the process $(\xi ^{\kappa }(t))_{t \ge 0}$ as given in Definition 1.2. We can estimate

$$\begin{aligned} q_{\kappa }:&=\max _{(n,m) \in E_{(n_0, m_0)}} \left\{ -(Q^{c_{\kappa }})_{(n,m),(n,m)}\right\} \le \left( {\begin{array}{c}n_0+m_0\\ 2\end{array}}\right) + c_{\kappa }(n_0+m_0) \\&\qquad + c_{\kappa }K(n_0+m_0). \end{aligned}$$

As we can see, condition (a) of Lemma 2.3 holds with

$$\begin{aligned} \frac{q_{\kappa }}{a_{\kappa }} = \frac{\left( {\begin{array}{c}n_0+m_0\\ 2\end{array}}\right) + c_{\kappa }(n_0+m_0) + c_{\kappa }K(n_0+m_0)}{c_{\kappa }^{-2}} \longrightarrow 0, \qquad \kappa \rightarrow \infty . \end{aligned}$$

Condition (b) was proven in Step (i). Therefore we may conclude

$$\begin{aligned} \left( N^{c_{\kappa }}(c^{-1}_{\kappa }t), M^{c_{\kappa }}(c^{-1}_{\kappa }t)\right) _{t \ge 0}&= \left( \xi ^{{\kappa }}\left( \frac{c^{-3}_{\kappa }}{c^{-2}_{\kappa }}t\right) \right) _{t \ge 0} \xrightarrow {\mathrm{f.d.d.}} \left( \tilde{N}(t), \tilde{M}(t)\right) _{t \ge 0} \end{aligned}$$

when $\kappa \rightarrow \infty$ and the proof of Theorem 1.5 is complete. $\square$

#### Remark 2.4

(*Imbalanced Island Size*) It is straightforward to pursue the same consideration for the *two-island model* and its *structured coalescent*Herbots (1994); Notohara (1990). The *two-island model* considers two populations much like the *seed bank model*, but allows for coalescence in the second population. Its genealogy is then given by the structured coalescent, whose block-counting process allows for the same transition rates described in (3) adding $$r_{(n,m),({\bar{n}},{\bar{m}})} = \left( {\begin{array}{c}m\\ 2\end{array}}\right) $$ for ${\bar{n}}=n$ and ${\bar{m}}=m-1$, i.e. coalescence in the second island (and adapting the diagonal entries accordingly).

Letting the migration rate converge $c\rightarrow 0$ while speeding up time by $1/c\rightarrow \infty$ as we have done for the block counting process of the seed bank coalescent above will lead to a structure with instantaneous coalescences in *both* islands, leaving us with a single line migrating between them.

In this set-up it is much more interesting to consider a two-island model with *different* scalings of the coalescence rates in the islands. In order to do this, we introduce the parameters $\alpha$ and $\alpha '$ such that the *Q*-matrix of the block-counting process of the structured coalescent now is

19 $$\begin{aligned} {\hat{R}}_{(n,m), ({\bar{n}},{\bar{m}})} = {\left\{ \begin{array}{ll} \alpha \left( {\begin{array}{c}n\\ 2\end{array}}\right) ,&{} \text {if } ({\bar{n}},{\bar{m}}) = (n-1,m),\\ \alpha '\left( {\begin{array}{c}m\\ 2\end{array}}\right) , &{} \text {if } ({\bar{n}},{\bar{m}}) = (n,m-1),\\ cn, &{} \text {if } ({\bar{n}},{\bar{m}}) = (n-1,m+1),\\ cKm, &{} \text {if } ({\bar{n}},{\bar{m}}) = (n+1,m-1),\\ 1 - \alpha \left( {\begin{array}{c}n\\ 2\end{array}}\right) - \alpha '\left( {\begin{array}{c}m\\ 2\end{array}}\right) - cn - cKm, &{} \text {if } ({\bar{n}},{\bar{m}}) = (n,m),\\ 0, &{} \text {otherwise.} \end{array}\right. } \end{aligned}$$

$\alpha$ and $\alpha '$ are associated with the notion of *effective population size* (cf. e.g. Wakeley 2009) so a different scaling corresponds to a significant difference in population size on the two islands. If, in addition to $c\rightarrow 0$ we assume the coalescence rate $\alpha '=\alpha '(c)>0$ in the second island to scale as *c*, i.e. $\alpha '/c \rightarrow 1$, the result is a two-island model with instantaneous coalescences in the first island, but otherwise ‘normal’ migration and coalescence behaviour in the second.

In order to formalise this heuristic observation, denote by $(N^{c,\alpha '}(t), M^{c,\alpha '}(t))_{t \ge 0}$ the block-counting process of the structured coalescent as defined by the rates in (19) with migration rate $c>0$ and coalescence rate $\alpha '>0$ in the second island and assume that it starts at some $(n_0, m_0) \in \mathbb {N}\times \mathbb {N}$, $\mathbb {P}$-a.s. (The parameters $\alpha , K>0$ are arbitrary but fixed.)

Define $({\hat{N}}(t), {\hat{M}}(t))_{t \ge 0}$ to be the continuous-time Markov chain with initial value $({\hat{N}}(0), {\hat{M}}(0))=(n_0, m_0)$, taking values in the state space $E_{(n_0,m_0)}:=\lbrace 0, \dots , n_0+m_0 \rbrace ^2$, with transition matrix $\Pi (t):=Pe^{t{\hat{G}}}$, for $t>0$ and $\Pi (0)=\mathrm {Id}_E$, where *P* is given by (7) (as in the case of seed banks) and ${\hat{G}}$ is now a matrix of the form

$$\begin{aligned} {\hat{G}}_{(n,m),({\bar{n}},{\bar{m}})} := {\left\{ \begin{array}{ll} Km + \left( {\begin{array}{c}m\\ 2\end{array}}\right) , &{} \text { if } {\bar{n}} = 1,\, n \ge 1,\, {\bar{m}} = m-1,\\ Km, &{} \text { if } {\bar{n}} = 1,\, n = 0,\, {\bar{m}} = m-1,\\ \left( {\begin{array}{c}m\\ 2\end{array}}\right) , &{} \text { if } {\bar{n}} = 0,\, n = 0,\, {\bar{m}} = m-1,\\ 1, &{} \text { if } {\bar{n}} = 0,\, n \ge 1,\, {\bar{m}} = m+1,\\ -\left( {\begin{array}{c}m\\ 2\end{array}}\right) - 1 - Km, &{} \text { if } {\bar{n}} = 1,\, n \ge 1,\, {\bar{m}} = m,\\ -\left( {\begin{array}{c}m\\ 2\end{array}}\right) - Km, &{} \text { if } {\bar{n}} = n = 0,\, {\bar{m}} = m,\\ 0, &{} \text { otherwise.} \end{array}\right. } \end{aligned}$$

Then, for any sequence of migration rates $(c_{\kappa })_{\kappa \in \mathbb {N}}$ and any sequence of coalescence rates $(\alpha '_{\kappa })_{\kappa \in \mathbb {N}}$ with $c_{\kappa } \rightarrow 0$ and $c_{\kappa }/\alpha '_{\kappa } \rightarrow 1$ when $\kappa \rightarrow \infty$

$$\begin{aligned} \left( N^{c_{\kappa }, \alpha '_{\kappa }}\left( \frac{1}{c_{\kappa }} t\right) , M^{c_{\kappa }, \alpha '_{\kappa }}\left( \frac{1}{c_{\kappa }} t\right) \right) _{t \ge 0} \xrightarrow {\mathrm{f.d.d.}} \big ({\hat{N}}(t), {\hat{M}}(t)\big )_{t \ge 0}, \quad \kappa \rightarrow \infty . \end{aligned}$$

This observation for the two-island model is analogous to Theorem 1.5 for seed banks. Its proof is a close parallel to that of Theorem 1.5. Considering, again, the sequences $a_{\kappa } := c^{-2}_{\kappa }$ and $b_{\kappa } := c^{-3}_{\kappa }$, $A_{\kappa }$ and *P* coincide with those in the proof of Theorem 1.5, hence the hardest work has already been done. Small alterations to $B_{\kappa }$ immediately yield the result and we therefore omit any further details.

## Scaling limits for the diffusion

We would now also like to observe similar scaling limits for the diffusion (2). As we saw in the case of Markov chains, rescaling time may lead to a limiting process that is still Markovian, but whose semi-group is not standard, i.e. not continuous in 0. We can use *moment duality* to obtain this limit.

### Convergence of the finite-dimensional distributions obtained from duality

We present a method to obtain convergence in finite-dimensional distributions of a sequence of Markov processes using moment duality and the convergence in finite-dimensional distributions of the dual processes. The result does not depend on whether time is rescaled, too, or not. It is, however, of particular interest in the rescaled case, since it might lead to the identification of limiting objects which rather “ill-behaved”. Indeed, we will see examples in Sect. 3.2 where the limit does not have a generator with a sufficiently large domain and hence the common approach of proving convergence through generator convergence fails.

For any tuples $n:=(n_1, \ldots , n_d) \in \mathbb {N}_0^d$ and $x:=(x_1, \ldots , x_d) \in [0,1]^d$, define the *mixed-moment function* $\mathfrak m$ as $\mathfrak m( x, n) := x_1^{n_1}\cdots x_d^{n_d}$.

#### Theorem 3.1

Let $(\zeta _{\kappa }(t))_{t\ge 0}$, $\kappa \in \mathbb {N}_0$, be a sequence of Feller processes taking values in $[0,1]^d$ (for some $d \in \mathbb {N}$), and $(\xi ^{\kappa }(t))_{t\ge 0}$, $\kappa \in \mathbb {N}_0$, a sequence of Markov chains with values in $\mathbb {N}_0^d$ such that they are pairwise moment duals, i.e.

$$\begin{aligned} \forall \kappa \in \mathbb {N}_0\;, \forall t\ge 0\; \forall x \in [0,1]^d, n \in \mathbb {N}_0^d:\; \mathbb {E}_n[\mathfrak m(x,\xi ^{\kappa }(t))]]=\mathbb {E}^x[\mathfrak m(\zeta _{\kappa }(t),n)]. \end{aligned}$$

As usual, $\mathbb {P}_n$ and $\mathbb {P}^x$ denote the distributions for which $\xi$ and $\zeta$, start in *n* and *x*, respectively.

If $(\xi ^{\kappa })_{\kappa \in \mathbb {N}_0}$ converges to some Markov chain $\xi$ in the f.d.d.-sense, then there exists a Markov process $\zeta$ with values in $[0,1]^d$ such that it is the f.d.d.-limit of $(\zeta _{\kappa })_{\kappa \in \mathbb {N}_0}$ and the moment dual to $\xi$, i.e.

20 $$\begin{aligned} \forall t\ge 0\; \forall x \in [0,1]^d, n \in \mathbb {N}_0^d:\; \mathbb {E}_n[\mathfrak m(x,\xi (t))]]=\mathbb {E}^x[\mathfrak m(\zeta (t),n)]. \end{aligned}$$

#### Remark 3.2

At first glance one might suspect that this result should also hold in a more general set-up as long as the employed duality function yields convergence determining families for the respective semi-groups. Indeed, most of the steps of the proof would still go through. However, note that we did not assume existence of a limiting Markov process beforehand. We can conclude this by the solvability of Hausdorff’s moment problem on $[0,1]^d$Hildebrandt and Schoenberg (1933), which precisely treats the existence (and uniqueness) of a distribution with a given sequence of *moments* and therefore “matches” the moment duality function in our theorem.

#### Proof of Theorem 3.1

The proof can roughly be split into three steps: We first use duality to prove the convergence of the *one-dimensional* distributions of $(\zeta _{\kappa })_{\kappa \in \mathbb {N}_0}$. This, together with the Markov property will give us the convergence of the *finite-dimensional distributions* of $(\zeta _{\kappa })_{\kappa \in \mathbb {N}_0}$ to a family of limiting distributions. Then we prove *consistency* of this family of distributions and hence by Kolmogorov’s Extension-Theorem the existence of a limiting *process* $\zeta$, which must then be Markovian.

Since the mixed-moment function $\mathfrak m$ is continuous and bounded as a function on $\mathbb {N}_0^d$, the convergence of the finite-dimensional distributions of $(\xi ^{\kappa })_{\kappa \in \mathbb {N}}$ and the assumed moment duality yield

21 $$\begin{aligned} \mathbb {E}^x[\mathfrak m(\zeta _{\kappa }(t),n)] = \mathbb {E}_n[\mathfrak m(x,\xi _{\kappa }(t))] \xrightarrow {\kappa \rightarrow \infty } \mathbb {E}_n[\mathfrak m(x,\xi (t))] =:\gamma (n,x,t) \end{aligned}$$

for any $t \ge 0$, $x \in [0,1]^d$ and $n \in \mathbb {N}_0^d$. For fixed $x \in [0,1]^d$ and $t\ge 0$ this is a monotonic sequence, i.e.

$$\begin{aligned} \forall n \in \mathbb {N}_0^d,\;\;\forall k_1 \le n_1, \ldots , k_d \le n_d: \quad \Delta ^{k_1}_1\cdots \Delta ^{k_d}_d \gamma (n,x,t) \ge 0, \end{aligned}$$

where $\Delta _i\gamma (n,x,t):=\gamma ((n_1, \ldots , n_{i-1}, n_i-1, n_{i+1}, \dots , n_l), x,t)-\gamma (n,x,t)$ is the difference operator acting on the *i*th component of *n*, $i = 1, \ldots , d$. This can be seen from (21):

22 $$\begin{aligned} \Delta ^{k_1}_1\cdots \Delta ^{k_d}_d \gamma (n,x,t)&= \lim _{\kappa \rightarrow \infty } \Delta ^{k_1}_1\cdots \Delta ^{k_d}_l \mathbb {E}_n[\mathfrak m(x,\xi _{\kappa }(t))] \nonumber \\&= \lim _{\kappa \rightarrow \infty } \Delta ^{k_1}_1\cdots \Delta ^{k_d}_l \mathbb {E}^x[\mathfrak m(\zeta _{\kappa }(t),n)] \nonumber \\&= \lim _{\kappa \rightarrow \infty } \mathbb {E}^x[\mathfrak m(\zeta _{\kappa }(t),n)(1-\zeta ^1_{\kappa }(t))^{k_1}\cdots (1-\zeta ^d_{\kappa }(t))^{k_d} ] \ge 0. \end{aligned}$$

Hence, the Hausdorff moment problem for $(\gamma (n,x,t))_{n \in \mathbb {N}_0^d}$ is solvable according to Theorem 1 in Hildebrandt and Schoenberg (1933), which means that there exists a measure $\mu ^{x,t}$ on $([0,1]^d, \mathfrak B([0,1]^d))$ (where $\mathfrak B$ is the Borel-$\sigma$-algebra) such that

$$\begin{aligned} \forall n \in \mathbb {N}^d_0:\quad \gamma (n,x,t) = \int _{[0,1]^d} \mathfrak m({\bar{x}},n){\text {d}}\mu ^{x,t}({\bar{x}}). \end{aligned}$$

In particular, this holds for $n=(0, \ldots , 0)$, hence $\mu ^{x,t}([0,1]^d) = \mathbb {E}_0[1] = 1$ and $\mu ^{x,t}$ is therefore a distribution. Since the polynomials are dense in the continuous functions, (21) implies the convergence of the one-dimensional distributions to $(\mu ^{x,t})_{t\ge 0}$ (for each starting point $x \in [0,1]^d$).

To check the convergence in finite-dimensional distributions, let us first make a general observation regarding weak convergence. Let $\mathcal P_{\kappa }$, $\kappa \in \mathbb {N}$, and $\mathcal P$ be distributions on $([0,1], \mathcal B([0,1]))$ such that the $\mathcal P_{\kappa }$ converge weakly to $\mathcal P$. Furthermore, let $f_{\kappa }, f:[0,1] \rightarrow \mathbb {R}$, $\kappa \in \mathbb {N}$, be continuous such that $f_{\kappa }(x)$ is uniformly bounded in $\kappa$ and *x* and converges to *f* pointwise (and therefore uniformly). Then we can estimate

23 $$\begin{aligned} \bigg \vert \int _{[0,1]^d}&f_{\kappa }(x) \mathcal P_{\kappa }(\mathrm{d}x) - \int _{[0,1]^d} f(x) \mathcal P(\mathrm{d}x) \bigg \vert \nonumber \\&\le \max _{x \in [0,1]^d} \left| f_{\kappa }(x) - f(x) \right| + \left| \int _{[0,1]^d} f(x) \mathcal P_{\kappa }(\mathrm{d}x)- \int _{[0,1]^d} f(x) \mathcal P(\mathrm{d}x) \right| \xrightarrow {\kappa \rightarrow \infty } 0 \nonumber \\ \end{aligned}$$

Returning to the task at hand, let $P_{\kappa }$ be the probability transition function of $\zeta _{\kappa }$ and recall that we assumed the $\zeta _{\kappa }$ to be Feller, hence $x \mapsto \int _{[0,1]^d} f({\bar{x}}) P_{\kappa }(x, t, \mathrm{d}{\bar{x}})$ is continuous and bounded by 1 for any *f* continuous and bounded by 1. For $0 \le t_1< \ldots< t_l <\infty$, $x \in [0,1]^d$ and $n^{(1)}, \ldots , n^{(l)} \in \mathbb {N}_0^d$ then observe

24 $$\begin{aligned} \mathbb {E}^x[&\mathfrak m(\zeta _{\kappa }(t_1),n^{(1)})\cdots \mathfrak m(\zeta _{\kappa }(t_l),n^{(l)})] \nonumber \\&= \int _{[0,1]^d}\cdots \int _{[0,1]^d}\mathfrak m({\bar{x}}^{(1)}, n^{(1)}) \cdots \mathfrak m({\bar{x}}^{(l)},n^{(l)})\nonumber \\&\qquad \qquad \qquad \qquad \qquad \qquad \qquad \qquad P_{\kappa }({\bar{x}}^{({l-1)}}, t_l-t_{l-1}, {\text {d}}{\bar{x}}^{(l)})\cdots P_{\kappa }(x, t_1, {\text {d}}{\bar{x}}^{(1)})\nonumber \\&= \int _{[0,1]^d}\mathfrak m({\bar{x}}^{(1)}, n^{(1)})\cdots \int _{[0,1]^d} \mathfrak m({\bar{x}}^{(l)},n^{(l)})P_{\kappa }({\bar{x}}^{({l-1)}}, t_l-t_{l-1}, {\text {d}}{\bar{x}}^{(l)})\cdots P_{\kappa }(x, t_1, {\text {d}}{\bar{x}}^{(1)})\nonumber \\&\xrightarrow { \kappa \rightarrow \infty }: \gamma (n^{(1)}, \ldots , n^{(l)},x,t_1, \ldots , t_l). \end{aligned}$$

Here we used the Markov property of $\zeta _{\kappa }$ in the first equality. For the convergence to some constant $\gamma (n^{(1)}, \ldots , n^{(l)},x,t_1, \ldots , t_l)$ we used the convergence of the finite-dimensional distributions shown above together with the observation that $x \mapsto \mathfrak m(x, n)$ is continuous and bounded by 1 (on $[0,1]^d$) and a recursive application of (23).

By the same argument as in (22), for fixed $x \in [0,1]^d$ and $t\ge 0$, $\gamma (n^{(1)}, \ldots , n^{(l)},x,t_1, \ldots , t_l)$, $(n^{(1)}, \ldots , n^{(l)}) \in (\mathbb {N}_0^d)^l$, is a monotonic sequence and Theorem 1 in Hildebrandt and Schoenberg (1933) yields the existence of a distribution $\mu ^{I,x}$ on $(([0,1]^d)^{I}, \mathfrak B([0,1]^d)^{\otimes I})$ for any finite set of indices $I=\{t_1, \ldots , t_l\} \subset [0,\infty )$ and starting point $x\in [0,1]^d$. In addition, (24) implies the convergence of the finite-dimensional distributions of $(\zeta _{\kappa })_{\kappa \in N}$ to a respective $\mu ^{I,x}$. Since these $\mu ^{I,x}$ are the limits of a consistent family they are themselves consistent and according to Kolmogorov’s Extension-Theorem there exists a unique measure $\mu ^x$ on the product-space $(([0,1]^d)^{[0,\infty )}, \mathfrak B([0,1]^d)^{\otimes [0,\infty )})$ which is the distribution of the desired process $\zeta$. This is a Markov process, because the $(\zeta _{\kappa })_{\kappa \in \mathbb {N}_0}$ are Markov processes.

The duality of $\zeta$ and $\xi$ follows from the duality of the prelimiting processes. $\square$

### Ancient ancestral material scaling regime

As an application of Theorem 3.1 we consider the diffusion (2) with the scaling regime of Sect. 2.2, namely, with the migration rate $c \rightarrow 0$ while simultaneously speeding up time by a factor $1/c \rightarrow \infty$ and obtain Theorem 1.3 stating the convergence of the rescaled diffusions to a Markovian limit $(\tilde{X}(t), \tilde{Y}(t))_{t \ge 0}$.

#### Theorem 3.3

Let $(X^{c}(t), Y^{c} (t))_{t \ge 0}$ be the seed bank diffusion given in Definition 1.1 with migration rate $c>0$. Assume that the initial distributions $(X^{c}(0), Y^{c}(0))$ converge weakly to a $(x,y) \in [0,1]^2$ as $c\rightarrow 0$. Then, there exists a Markov process $(\tilde{X}(t),\tilde{Y}(t))_{t\ge 0}$, started in $(\tilde{X}(0),\tilde{Y}(0))=(x,y)$ with the property that for any sequence of migration rates with $c_{\kappa }\rightarrow 0$ when $\kappa \rightarrow \infty$,

$$\begin{aligned} \left( X^{c_{\kappa }}\left( \frac{1}{c_{\kappa }}t\right) , Y^{c_{\kappa }} \left( \frac{1}{c_{\kappa }}t\right) \right) _{t \ge 0} \xrightarrow {\mathrm{f.d.d.}} (\tilde{X}(t),\tilde{Y}(t))_{t\ge 0} \quad \text {as }\kappa \rightarrow \infty \end{aligned}$$

and $(\tilde{X}(t),\tilde{Y}(t))_{t\ge 0}$ is the moment dual of $(\tilde{N}(t),\tilde{M}(t))_{t \ge 0}$ given in Definition 1.4.

Note that $(\tilde{X}(t),\tilde{Y}(t))_{t\ge 0}$ makes sense for more general initial conditions in $[0,1]^2$. In any case, the limiting process $(\tilde{X}(t),\tilde{Y}(t))_{t\ge 0}$ instantaneously jumps into the smaller state space $\{0,1\}\times [0,1]$ at time $0+$. The jump probabilities to 0 and 1 are the fixation probabilities of the ordinary Wright-Fisher diffusion. This corresponds to an instantaneous application of a projection operator $\tilde{P}$ defined as the limit (in a suitable sense)

$$\begin{aligned} \tilde{P}:= \lim _{t \rightarrow \infty } \tilde{P}_t, \end{aligned}$$

where $(\tilde{P}_t)_{t\ge 0}$ is the semi-group associated to the classical Wright-Fisher diffusion, cf. Kurtz (1973) (or Bobrowski 2015, Equation (3)). Intuitively, this can be explained as follows: In the regime, where dormancy duration is significantly larger than the effect of genetic drift, the population evolves according to a Wright-Fisher diffusion without dormancy and has the chance to be absorbed in 0 or 1, before ever seeing a resuscitation/migration into the population from the seed bank. Hence, on the super-evolutionary time-scale the probabilitiesIntuitively to immediately jump to 0 or 1 are precisely given by the corresponding fixation probabilities of the Wright-Fisher diffusion.

#### Remark 3.4

(*Convergence on path space?*) Once convergence of the finite-dimensional distributions is established in Theorem 1.3, it is natural (at least for mathematicians) to ask whether it is possible to prove tightness on the space of càdlàg paths space in order to obtain weak convergence. However, since the set of continuous paths form a closed subset of the càdlàg paths in the classical Skorohod ($J_1$) topology (cf. Skorohod (1956)), and the solutions to our pre-limiting seed bank diffusions *are* continuous, convergence in the above topologies would predict a limit with continuous paths, which we know not to be correct at least in 0. This makes weak convergence on path space impossible. However, the set of jump times of the above process is finite on finite time intervals, and in particular has Lebesgue-measure zero, so that we expect that convergence is true in weaker topologies, such as the Meyer-Zheng topology corresponding to convergence in measure (Meyer and Zheng 1984; Kurtz 1991). However, we refrain from going into these technicalities here, which we consider to be outside the scope of this manuscript.

#### Remark 3.5

Remark 3.2 in Shiga and Shimizu (1980) implies that the unique strong solution to the SDE (1.1) which is the seed bank diffusion from Definition 2 is a Feller process. This is considered in more generality in Theorem 2.4 in Greven et al. (2020).

#### Proof of Theorem 3.3

Since the $\left( X^{c_{\kappa }}\left( t/c_{\kappa }\right) , Y^{c_{\kappa }} \left( t/c_{\kappa }\right) \right) _{t \ge 0}$ are constant time-changes of the seed bank diffusion introduced in Definition 1.1, they are Feller, as well.

Since the moment duality of the block-counting process of the seed bank coalescent and the seed bank diffusion (4) holds for every time $t\ge 0$, it is preserved for the time-changed processes $\left( N^{c_{\kappa }}\left( t/c_{\kappa }\right) , M^{c_{\kappa }} \left( t/c_{\kappa }\right) \right) _{t \ge 0}$ and $\left( X^{c_{\kappa }}\left( t/c_{\kappa }\right) , Y^{c_{\kappa }} \left( t/c_{\kappa }\right) \right) _{t \ge 0}$. Together with Theorem 1.5 all assumptions of Theorem 3.1 hold and we get the existence of a Markov process $(\tilde{X}(t),\tilde{Y}(t))_{t\ge 0}$ that is the dual of $(\tilde{N}(t),\tilde{M}(t))_{t\ge 0}$. By the uniqueness of the solution to the Hausdorff moment problem (Theorem 2 in in Hildebrandt and Schoenberg 1933) a distribution on $[0,1]^2$ is uniquely determined by all its mixed-moments. The moment duality of the limit with a process that does not depend on the scaling sequence $(c_{\kappa })_{\kappa \in \mathbb {N}_0}$ therefore implies that the one-dimensional distributions of the limit do not depend on the choice of scaling sequence, either. Since the limit is a Markov-process the one-dimensional distributions uniquely determine its entire distribution. Hence, the distribution of the limit does not depend on the choice of scaling sequence $(c_{\kappa })_{\kappa \in \mathbb {N}_0}$. $\square$

So far we have characterised the process $$(\tilde{X}(t),\tilde{Y}(t))_{t\ge 0}$$ only as the moment dual of the continuous-time Markov chain $$(\tilde{N}(t),\tilde{M}(t))_{t\ge 0}$$ whose semi-group we could give explicitly in Definition 1.4. We now use this characterisation to better understand the process $$(\tilde{X}(t),\tilde{Y}(t))_{t\ge 0}$$ itself. More precisely, since (20) holds in particular for $$t >0 $$, $$m=0$$ and any $$n\ge 1$$, $$x,y \in [0,1]$$ we see

25 $$\begin{aligned} \mathbb {E}^{x,y}&[\tilde{X}(t)^n\tilde{Y}(t)^0] = \mathbb {E}_{n,0}[x^{\tilde{N}(t)}y^{\tilde{M}(t)}] \nonumber \nonumber \\= & {} x\mathbb {P}_{n,0}(\tilde{N}(t) = 1, \tilde{M}(t) = 0) + y\mathbb {P}_{n,0}(\tilde{N}(t) = 0, \tilde{M}(t) = 1) \nonumber \\= & {} x (Pe^{tG})_{(n,0),(1,0)}+y (Pe^{tG})_{(n,0),(0,1)} = x (e^{tG})_{(1,0),(1,0)} + y(e^{tG})_{(1,0),(0,1)}. \nonumber \\ \end{aligned}$$

We used the fact that the first component of the ancient ancestral lines process $(\tilde{N}(t),\tilde{M}(t))_{t\ge 0}$ takes values in $\{0,1\}$ for any $t>0$ in the second equality and the definition of the projection in the last equality. Since the right-hand side does not depend on $n \ge 1$, we can conclude that

26 $$\begin{aligned} \tilde{X}(t) \in \{0,1\}\quad \mathbb {P}^{x,y}\text {-a.s.\ for any } t>0 \text { and any }(x,y)\in [0,1]^2. \end{aligned}$$

We can use this observation together with (25) to obtain

27 $$\begin{aligned} \lim _{t\downarrow 0}\mathbb {P}^{x,y}\{\tilde{X}(t) = 1\} = \lim _{t\downarrow 0} \mathbb {E}^{x,y}[\tilde{X}(t)^n] = x(\mathrm{I}_{\mathbb {N}_0^2})_{(1,0),(1,0)} + y(\mathrm{I}_{\mathbb {N}_0^2})_{(1,0),(0,1)} = x. \end{aligned}$$

(Here $(\mathrm{I}_{\mathbb {N}_0^2})$ is the identity matrix on $\mathbb {N}_0^2$.)

This small observation has an important consequence: Much like in the case of its dual $(\tilde{N}(t), \tilde{M}(t))_{t \ge 0}$, the semi-group of the ancient ancestral material process $(\tilde{X}(t), \tilde{Y}(t))_{t \ge 0}$ is not right-continuous in 0.

Intuitively, the reproduction mechanism (in the active population) acts so fast, that fixation (or extinction) *in the active population* happens instantaneously. Whenever there is an invasion from the seed bank, the chances that this is by an individual of the type extinct in the active population (thereby causing a change of type here) are given by the frequency of said type in the *dormant* population. The limit is thus a pure jump process in the active component that moves between the states 0 and 1 at rates proportional to the frequency in the *dormant* population of the allele that is extinct in the *active* population, while the seed bank component retains its classical behaviour. We can formalise this observation if we restrict the process to the smaller state space $\{0,1\}\times [0,1]$, see Proposition 3.8 below.

#### Definition 3.6

Let $({\bar{N}}(t), {\bar{M}}(t))_{t \ge 0}$ be the Markov chain on $\{0,1\}\times \mathbb {N}_0$ given by the *Q*-matrix

$$\begin{aligned} {\bar{G}}_{(n,m),({\bar{n}},{\bar{m}})} = {\left\{ \begin{array}{ll} Km, &{} \text { if } {\bar{n}} = 1,\, n \in \{0,1\},\, {\bar{m}} = m-1,\\ 1, &{} \text { if } {\bar{n}} = 0,\, n = 1,\, {\bar{m}} = m+1,\\ -n-Km, &{} \text { if } {\bar{n}} = n ,\, {\bar{m}} = m,\\ 0, &{} \text { otherwise.} \end{array}\right. } \end{aligned}$$

for any $$(n,m),({\bar{n}},{\bar{m}}) \in \{0,1\}\times \mathbb {N}_0$$.

Furthermore, let $({\bar{X}}(t), {\bar{Y}}(t))_{t \ge 0}$ be the Markov process on $\{0,1\}\times [0,1]$ defined by the generator given in (6).

#### Proposition 3.7

$({\bar{X}}(t), {\bar{Y}}(t))_{t \ge 0}$ is well-defined i.e. the closure of $\bar{\mathcal A}$ given in (6) is indeed the generator of a Markov process and this process is Feller. Furthermore, we may assume that $({\bar{X}}(t), {\bar{Y}}(t))_{t \ge 0}$ is cádlág on $[0,\infty )$.

#### Proof

Define $$E:=\{0,1\}\times [0,1]$$ and

$$\begin{aligned} \mathcal C&:=\mathcal C(E, \mathbb {R}) =\{f:E\rightarrow \mathbb {R}\mid f(0,\cdot ), f(1, \cdot ): [0,1]\rightarrow \mathbb {R}\; \text {are continuous}\},\\ \mathcal D_{\bar{\mathcal A}}:=\mathcal C^1&:=\{f:E\rightarrow \mathbb {R}\mid f(0,\cdot ), f(1, \cdot ): [0,1]\rightarrow \mathbb {R}\; \text {are differentiable}\}. \end{aligned}$$

We verify the conditions of the Hille–Yosida Theorem, cf. Theorem 19.11 in Kallenberg (2002), for $(\bar{\mathcal A}, \mathcal D_{\bar{\mathcal A}})$, where $\bar{\mathcal A}$ is given in (6). First note that

$$\begin{aligned} \{f:E\rightarrow \mathbb {R}\mid f(0,\cdot ), f(1, \cdot ): [0,1]\rightarrow \mathbb {R}\; \text {are polynomials}\} \subset \mathcal D_{\bar{\mathcal A}} \end{aligned}$$

hence $\mathcal D_{\bar{\mathcal A}}$ is dense in $\mathcal C$. In order to verify the maximum principle choose an arbitrary $f \in \mathcal D_{\bar{\mathcal A}}$ and let $({\bar{x}}, {\bar{y}}) \in E$ be such that $f({\bar{x}}, {\bar{y}}) \ge f(x,y)\wedge 0$ for all $(x,y)\in E$. Then

$$\begin{aligned} \mathcal Af({\bar{x}},{\bar{y}})&= (1-{\bar{x}}){\bar{y}}(f(1,{\bar{y}}) - f({\bar{x}},{\bar{y}})) + {\bar{x}}(1-{\bar{y}})(f(0,{\bar{y}}) - f({\bar{x}}, {\bar{y}}))\\&\qquad + K({\bar{x}} - {\bar{y}})\frac{\partial f}{\partial y}({\bar{x}}, {\bar{y}}) \end{aligned}$$

Since we assumed *f* to have a maximum in $({\bar{x}}, {\bar{y}})$, the first two summands are non-positive. If ${\bar{y}} \in (0,1)$, a maximum in $({\bar{x}}, {\bar{y}})$ implies $\frac{\partial f}{\partial y}({\bar{x}}, {\bar{y}}) =0$. If ${\bar{y}} = 0$, a maximum in $({\bar{x}}, {\bar{y}})$ implies $\frac{\partial f}{\partial y}({\bar{x}}, {\bar{y}}) \le 0$ and therefore $({\bar{x}} - 0)\frac{\partial f}{\partial y}({\bar{x}}, {\bar{y}}) \le 0$. Likewise, if ${\bar{y}} = 1$, a maximum in $({\bar{x}}, {\bar{y}})$ implies $\frac{\partial f}{\partial y}({\bar{x}}, {\bar{y}}) \ge 0$ and therefore $({\bar{x}} - 1)\frac{\partial f}{\partial y}({\bar{x}}, {\bar{y}}) \le 0$. Hence, $\bar{\mathcal A}f({\bar{x}}, {\bar{y}}) \le 0$ and the maximum principle holds. We are left to prove that there exists a $\lambda >0$ such that $(\lambda - \bar{\mathcal A})\mathcal D_{\bar{\mathcal A}}$ is dense in $\mathcal C$. First, observe that $f \in \mathcal C$ if and only if it can be written in the form $f(x,y) = (1-x)f_0(y) + xf_1(y)$, where $f_0(\cdot ), f_1(\cdot ): [0,1]\rightarrow \mathbb {R}$ are continuous. Since the polynomials are dense in the continuos functions on [0, 1] and $\bar{\mathcal A}$ is a linear operator, it suffices to show that for any $r \in \mathbb {N}_0$ we can find $f_{(r)}, f^{(r)}\in \mathcal D_{\bar{\mathcal A}}$ such that $(\lambda - \bar{\mathcal A})f_{(r)}(x,y) = (1-x)y^r$ and $(\lambda - \bar{\mathcal A})f^{(r)}(x,y) = xy^r$. In an intuitive abuse of notation, we will in the following denote maps of the form $(x,y) \mapsto xy^r$ by $xy^r$ and likewise for $(1-x)y^r$. We begin by calculating, for any $r \in \mathbb {N}_0$

$$\begin{aligned} (\lambda - \bar{\mathcal A})xy^r&= \lambda xy^r - (1-x)y(y^r - xy^r) - x(1-y)(0-xy^r) - K(x-y)xry^{r-1}\\&= (1-x)\big (-y^{r+1}\big ) + x\big (-rKy^{r-1} + (\lambda + 1 + rK)y^r -y^{r+1}\big ),\\&= x\big (-rKy^{r-1} + (\lambda + 1 + rK)y^r) -y^{r+1}\\ (\lambda - \bar{\mathcal A})(1-x)y^r&= \lambda (1-x)y^r - (1-x)y(0 - (1-x)y^r) \\&\qquad \qquad \qquad - x(1-y)(y^r-(1-x)y^r) - K(x-y)(1-x)ry^{r-1}\\&= (1-x)\big ((\lambda +rK)y^r+y^{r+1}\big )\big ) + x\big (-y^r + y^{r+1}\big ),\\&= (1-x)(\lambda +rK)y^r + x\big (-y^r\big ) + y^{r+1} \\ (\lambda - \bar{\mathcal A})y^r&= x\big (-rKy^{r-1}\big ) + (\lambda + rK)y^r. \end{aligned}$$

Proceed by induction on the degree *r*, beginning with $r=0$. Observe that $(\lambda - \bar{\mathcal A})1 = \lambda$ and

$$\begin{aligned} (\lambda - \bar{\mathcal A})\left\{ (1-x) - \frac{1}{\lambda +K}y\right\}&= (1-x)\lambda - x + y - \frac{1}{\lambda +K}\left( -xK + (\lambda +K)y\right) \\&= (1-x)\lambda + x\frac{-\lambda }{\lambda + K}, \end{aligned}$$

therefore

$$\begin{aligned}&(\lambda - \bar{\mathcal A})\frac{\lambda +K}{-\lambda (\lambda +K+1)}\left\{ (1-x) - \frac{1}{\lambda +K}y-1\right\} \\&\quad = \frac{\lambda +K}{-\lambda (\lambda +K+1)}\left\{ x(-\lambda ) + x\frac{-\lambda }{\lambda + K} \right\} =xy^0 \end{aligned}$$

and immediately also

$$\begin{aligned} (\lambda - \bar{\mathcal A})\left\{ \frac{1}{\lambda } - \frac{\lambda +K}{-\lambda (\lambda +K+1)}\left\{ (1-x) - \frac{1}{\lambda +K}y-1\right\} \right\}&= 1-x = (1-x)y^0. \end{aligned}$$

Now let $n \in \mathbb {N}$ and assume that for any $r \le n-1$ there exist $f_{(r)}, f^{(r)}\in \mathcal D_{\bar{\mathcal A}}$ such that $(\lambda - \bar{\mathcal A})f_{(r)}(x,y) = (1-x)y^r$ and $(\lambda - \bar{\mathcal A})f^{(r)}(x,y) = xy^r$. Note that

$$\begin{aligned} (\lambda - \bar{\mathcal A})\left\{ y^n + nKf^{(n-1)}(x,y)\right\}&= (\lambda +nK) y^n. \end{aligned}$$

In addition, similarly to the above,

$$\begin{aligned}&(\lambda - \bar{\mathcal A})\left\{ (1-x)y^n - \frac{1}{\lambda + (n+1)K}y^{n+1}\right\} \\&\quad = (1-x)(\lambda + nK)y^n + x\frac{-\lambda }{\lambda + (n + 1)K}y^n. \end{aligned}$$

Hence we may again obtain

$$\begin{aligned} (\lambda - \bar{\mathcal A})&\frac{-\lambda - (n + 1)K}{\lambda + (\lambda + nK)(\lambda + (n + 1)K)}\\&\qquad \qquad \qquad \qquad \times \left\{ (1-x)y^n - \frac{1}{\lambda + (n + 1)K}y^{n+1} - \left( y^n + nKf^{(n-1)}\right) \right\} \\&= \frac{-\lambda - (n + 1)K}{\lambda + (\lambda + nK)(\lambda + (n + 1)K)}\left\{ x(\lambda +nK)(-y^n) + x\frac{-\lambda }{\lambda + (n + 1)K}y^n\right\} \\&= xy^n \end{aligned}$$

and with this also

$$\begin{aligned} (\lambda - \bar{\mathcal A})&\Bigg \{\frac{1}{\lambda +nK}\left\{ y^n + nKf^{(n-1)}(x,y)\right\} \\&\quad + \frac{\lambda + (n + 1)K}{\lambda + (\lambda + nK)(\lambda + (n + 1)K)}\\&\qquad \qquad \qquad \qquad \times \left\{ (1-x)y^n - \frac{1}{\lambda + (n + 1)K}y^{n+1} - \left( y^n + nKf^{(n-1)}\right) \right\} \Bigg \} \\&= y^n - xy^n = (1-x)y^n. \end{aligned}$$

This completes the proof that $(\lambda - \bar{\mathcal A})\mathcal D_{\bar{\mathcal A}}$ is dense in $\mathcal C$. Hence, the closure of $\bar{\mathcal A}$ generates a Feller semigroup on $\mathcal C$. According to Kallenberg (2002, Proposition 19.14) this Feller semigroup then generates a Feller process, which we may assume to be cádlág paths thanks to Kallenberg (2002, Theorem 19.15).

Both processes correspond to the ancient ancestral material scaling when considering only the reduced “effective” state spaces:

#### Proposition 3.8

The processes $({\bar{N}}(t), {\bar{M}}(t))_{t \ge 0}$ and $({\bar{X}}(t), {\bar{Y}}(t))_{t \ge 0}$ introduced in Definition 3.6 are moment duals, i.e.

28 $$\begin{aligned} \forall t\ge 0\; \forall (x,y) \in [0,1]^2, (n,m) \in \mathbb {N}_0^2:\; \mathbb {E}_{n,m}\left[ x^{{\bar{N}}(t)}y^{{\bar{M}}(t)}\right] =\mathbb {E}^{x,y}\left[ {{\bar{X}}(t)}^n{{\bar{Y}}(t)}^m\right] . \end{aligned}$$

Furthermore, $({\bar{N}}(t), {\bar{M}}(t))_{t \ge 0}$ coincides in distribution with $(\tilde{N}(t), \tilde{M}(t))_{t \ge 0}$ if (both are) started in the reduced state-space $\{0,1\}\times \mathbb {N}_0$.

Likewise, $({\bar{X}}(t), {\bar{Y}}(t))_{t \ge 0}$ coincides in distribution with $(\tilde{X}(t), \tilde{Y}(t))_{t \ge 0}$ if (both are) started in the reduced state-space $\{0,1\}\times [0,1]$.

Moment duality of the involved processes will be important for the proof of the last statement, which is crucial for the proof of Theorem 1.3.

**Fig. 4:**
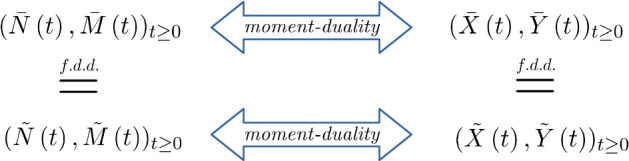
Strategy of the proof of Proposition 3.8. The moment duality of $(\tilde{N}(t), \tilde{M}(t))_{t \ge 0}$ and $(\tilde{X}(t), \tilde{Y}(t))_{t \ge 0}$ is a consequence of Theorem 3.3. The laws of $(\tilde{N}(t), \tilde{M}(t))_{t \ge 0}$ and $({\bar{N}}(t), {\bar{M}}(t))_{t \ge 0}$ agree when restricted to the reduced state-space $\{0,1\}\times \mathbb {N}_0$. We show the moment duality of $({\bar{N}}(t), {\bar{M}}(t))_{t \ge 0}$ and $({\bar{X}}(t), {\bar{Y}}(t))_{t \ge 0}$, which then allows us to conclude that the restricted laws of $(\tilde{X}(t), \tilde{Y}(t))_{t \ge 0}$ and $({\bar{X}}(t), {\bar{Y}}(t))_{t \ge 0}$ also agree on $\{0,1\}\times [0,1]$

#### Proof

We prove the claims in order of appearance.

The duality of $({\bar{N}}(t),{\bar{M}}(t))_{t\ge 0}$ and $({\bar{X}}(t),{\bar{Y}}(t))_{t\ge 0}$ can be shown using the respective generators: Define $S((x,y),(n,m)):=S^{x,y}(n,m):=S_{n,m}(x,y):=x^ny^m$ for $(n,m) \in \{0,1\}\times \mathbb {N}_0$ and $(x,y) \in \{0,1\}\times [0,1]$. Applying $\bar{\mathcal A}$ to $S_{n,m}$ yields

$$\begin{aligned} \bar{\mathcal A} S_{n,m}(x,y)&= y(y^m-0^ny^m)1\mathrm {l}_{\{0\}}(x) + (1-y)(0^ny^m-y^m)1\mathrm {l}_{\{1\}}(x)\\&\qquad + K(x-y)x^nmy^{m-1}\\&= -(x-y)(y^m-0^ny^m)1\mathrm {l}_{\{0\}}(x) + (x-y)(0^ny^m-y^m)1\mathrm {l}_{\{1\}}(x)\\&\qquad + K(x-y)x^nmy^{m-1}\\&= -n(x-y)y^m + Km(x-y)x^ny^{m-1}\\&= Kmx^{n+1}y^{m-1} + (-Kmx^n - nx)y^m + ny^{m+1} \end{aligned}$$

where we continue to use $0^0=1$, the fact that $n \in \{0,1\}$ and simply sorted the terms by powers of *y* for easier comparison in the last line.

In order to do the analogous calculation for $({\bar{N}}(t),{\bar{M}}(t))_{t\ge 0}$ we need its generator. Since ${\bar{G}}$ is the conservative *Q*-matrix, the generator $\mathcal G$ is given by

$$\begin{aligned} \mathcal {{\bar{G}}} f(n,m)&:= \sum _{({\bar{n}}, {\bar{m}}) \in \{0,1\}\times \mathbb {N}_0} {\bar{G}}_{(n,m),({\bar{n}}, {\bar{m}})}f({\bar{n}}, {\bar{m}})\\&= Km(f(1, m-1) - f(n,m)) + (f(0,m+1) - f(n,m))1\mathrm {l}_{\{1\}}(n) \end{aligned}$$

for all $f:\{0,1\}\times \mathbb {N}_0 \rightarrow \mathbb {R}$ which are bounded. If we apply $\mathcal G$ to *S* as a function in $(n,m)\in \{0,1\}\times \mathbb {N}_0$, we get

$$\begin{aligned} \mathcal {{\bar{G}}} S^{x,y}(n,m)&= Km(xy^{m-1}-x^ny^m) + (y^{m+1}-xy^m)1\mathrm {l}_{\{1\}}(n) \\&= Km(xy^{m-1}-xy^m)\underbrace{1\mathrm {l}_{\{1\}}(n)}_{=n} + Km(xy^{m-1}-y^m)\underbrace{1\mathrm {l}_{\{0\}}(n)}_{=1-n} \\&\qquad + 1(y^{m+1}-xy^m)\underbrace{1\mathrm {l}_{\{1\}}(n)}_{=n}\\&= Kmxy^{m-1} + (-Kmnx -Km(1-n)-nx)y^m + ny^{m+1}. \end{aligned}$$

A close look noting that for our choices of variables we have $x^{n+1}=x$ and $nx+(1-n) = x^n$ shows that

$$\begin{aligned} \forall (x,y) \in \{0,1\}\times [0,1],\;(n,m) \in \{0,1\}\times \mathbb {N}_0: \quad \bar{\mathcal A} S_{n,m}(x,y) = \mathcal {{\bar{G}}} S^{x,y}(n,m). \end{aligned}$$

*S* is bounded and continuous. For any $t \ge 0$, $(x,y) \in \{0,1\}\times [0,1]$ the functions $S^{x,y}$ and $\{0,1\}\times \mathbb {N}_0 \ni (n,m)\mapsto \mathbb {E}^{x,y}[{\bar{X}}(t)^n{\bar{Y}}(t)^m]$ are bounded. Furthermore, for any $t\ge 0$, $(n,m)\in \{0,1\}\times \mathbb {N}_0$, the functions $S_{n,m}$ and $\{0,1\}\times [0,1] \ni (x,y) \mapsto \mathbb {E}_{n,m}[x^{\tilde{N}(t)}y^{\tilde{M}(t)}]$ are continuously differentiable on (0, 1) and continuous on [0, 1] in the second component and continuous due to the theorem of bounded convergence. Hence, all assumptions of Jansen and Kurt (2014, Prop. 1.2) hold and we have proven the duality.

Next, we want to prove the equality of $(\tilde{N}(t),\tilde{M}(t))_{t\ge 0}$ and $({\bar{N}}(t),{\bar{M}}(t))_{t\ge 0}$ in distribution, if both processes have the same initial distribution (which then must be in the smaller space $\{0,1\}\times \mathbb {N}_0)$). Recall that $(Pe^{tG})_{t \ge 0}$ is the semi-group of $(\tilde{N}(t),\tilde{M}(t))_{t\ge 0}$ from Definition 1.4. On the other hand, the semi-group of $({\bar{N}}(t),{\bar{M}}(t))_{t\ge 0}$ is given by $(e^{t{\bar{G}}})_{t\ge 0}$. Since both are Markov processes it suffices to prove that they both have the same semi-group. Note that technically these semi-groups have different dimensions, so to be precise, we want to prove that the restriction of $(Pe^{tG})_{t \ge 0}$ to the space $\{0,1\}\times \mathbb {N}_0$ coincides with $(e^{t{\bar{G}}})_{t\ge 0}$, i.e.

$$\begin{aligned} \forall t \ge 0:\quad ((Pe^{tG})_{i,j})_{i,j \in \{0,1\}\times \mathbb {N}_0} = e^{t{\bar{G}}}. \end{aligned}$$

This will be true, because of the structure of *G* that reflects that the space $\{0,1\}\times \mathbb {N}_0$ is absorbing for $(\tilde{N}(t),\tilde{M}(t))_{t\ge 0}$. More precisely, we prove by induction that

29 $$\begin{aligned} (G^k_{i,j})_{i,j \in \{0,1\}\times \mathbb {N}_0} = {\bar{G}}^k \end{aligned}$$

for all $k \in \mathbb {N}$. Comparing the definitions of *G* in Definition 1.4 and of ${\bar{G}}$ in Definition 3.6, we see that (29) holds for $k=1$. Assume this is true for some fixed $k \in \mathbb {N}$. Then, for $(n,m),({\bar{n}}, {\bar{m}}) \in \{0,1\}\times \mathbb {N}_0$

$$\begin{aligned} G^{k+1}_{(n,m),({\bar{n}}, {\bar{m}})}&= \sum _{(l_1, l_2) \in \mathbb {N}_0^2} G^{1}_{(n,m),(l_1, l_2)}G^{k}_{(l_1, l_2),({\bar{n}}, {\bar{m}})} \\&\overset{*}{=} \sum _{(l_1, l_2) \in \{0,1\}\times \mathbb {N}_0} \underbrace{G^{1}_{(n,m),(l_1, l_2)}}_{= {\bar{G}}^{1}_{(n,m),(l_1, l_2)}}\;\underbrace{G^{k}_{(l_1, l_2),({\bar{n}}, {\bar{m}})}}_{= {\bar{G}}^{k}_{(l_1, l_2),({\bar{n}}, {\bar{m}})}} = {\bar{G}}^{k+1}_{(n,m),({\bar{n}}, {\bar{m}})} \end{aligned}$$

where we used that $G_{(n,m),(l_1, l_2)} = 0$ if $(l_1, l_2) \notin \{0,1\}\times \mathbb {N}_0$ in $*$ and then applied the induction assumption. Hence (29) does indeed hold for any $k \in \mathbb {N}$. Hence, for every choice of $(n,m), ({\bar{n}}, {\bar{m}}) \in \{0,1\}\times \mathbb {N}_0$ and $t \ge 0$, recalling that $PG = G$,

$$\begin{aligned} (Pe^{tG})_{(n,m),({\bar{n}}, {\bar{m}})}&= \sum _{k \in \mathbb {N}_0} \frac{t^k}{k!} G^k_{(n,m),({\bar{n}}, {\bar{m}})} = \sum _{k \in \mathbb {N}_0} \frac{t^k}{k!} {\bar{G}}^k_{(n,m),({\bar{n}}, {\bar{m}})} = (e^{t{\bar{G}}})_{(n,m),({\bar{n}}, {\bar{m}})}. \end{aligned}$$

Therefore the processes $(\tilde{N}(t),\tilde{M}(t))_{t\ge 0}$ and $({\bar{N}}(t),{\bar{M}}(t))_{t\ge 0}$ do indeed coincide in distribution if started in the same state $(n,m) \in \{0,1\}\times \mathbb {N}_0$.

Since we now in particular have the equality of the one-dimensional distributions, we can use the duality (28) and the duality given in Theorem 3.3 to obtain

30 $$\begin{aligned} \mathbb {E}^{x,y}\left[ {\tilde{X}(t)}^n{\tilde{Y}(t)}^m\right] =\mathbb {E}_{n,m}\left[ x^{\tilde{N}(t)}y^{\tilde{M}(t)}\right] =\mathbb {E}_{n,m}\left[ x^{{\bar{N}}(t)}y^{{\bar{M}}(t)}\right] =\mathbb {E}^{x,y}\left[ {{\bar{X}}(t)}^n{{\bar{Y}}(t)}^m\right] \end{aligned}$$

for all $t\ge 0$ and all $(x,y) \in \{0,1\}\times [0,1]$ and $(n,m) \in \{0,1\}\times \mathbb {N}_0$.

Recall from (26) that for any $t>0$ we have $(\tilde{X}(t),\tilde{Y}(t)) \in \{0,1\}\times [0,1]$, $\mathbb {P}^{x,y}$-a.s., $(x,y) \in [0,1]^2$. Since a distribution on $\{0,1\}\times [0,1]$ is uniquely determined by its moments of order $(n,m)\in \{0,1\}\times \mathbb {N}_0$, (30) implies that $(\tilde{X}(t),\tilde{Y}(t)) \sim ({\bar{X}}(t),{\bar{Y}}(t))$ for any $t>0$ (when started in the same $(x,y) \in \{0,1\}\times [0,1]$). Since they are both Markovian, this implies that the distributions of $({\bar{X}}(t), {\bar{Y}}(t))_{t \ge 0}$ and $(\tilde{X}(t), \tilde{Y}(t))_{t \ge 0}$ coincide when started in the reduced state-space $\{0,1\}\times [0,1]$. $\square$

Combining these results we obtain the proof of Theorem 1.3.

#### Proof of Theorem 1.3

Theorem 3.3 already yields the existence of $(\tilde{X}(t),\tilde{Y}(t))_{t \ge 0}$ as the limit in finite-dimensional distributions.

(5) is simply the observation of (27).

Hence we are left to prove that we can choose a process with the above properties (determined only by the distribution!) with nice path-properties.

Fix, $(x,y) \in [0,1]^2$. Now, let $({\bar{X}}_*(t), {\bar{Y}}_*(t))_{t \ge 0}$ and $({\bar{X}}^*(t), {\bar{Y}}^*(t))_{t \ge 0}$ be independent copies of $({\bar{X}}(t), {\bar{Y}}(t))_{t \ge 0}$, starting at $(0,y) \in \{0,1\}\times [0,1]$ and $(1,y) \in \{0,1\}\times [0,1]$, respectively. Furthermore, let *B* be an independent Bernoulli random variable with success parameter *x*. With this, define the process

$$\begin{aligned} \forall t\ge 0: \quad (\tilde{\tilde{X}}(t),\tilde{ \tilde{ Y}}(t)):= B({\bar{X}}_*(t), {\bar{Y}}_*(t))_{t \ge 0} + (1-B)({\bar{X}}^*(t), {\bar{Y}}^*(t))_{t \ge 0}. \end{aligned}$$

This process is cádlág (with a random initial distribution (*B*, *y*)). We now prove that $(\tilde{X}(t),\tilde{Y}(t))_{t>0}$ and $(\tilde{\tilde{X}}(t),\tilde{\tilde{Y}}(t))_{t>0}$ are equal in distribution. (Note that we claim this for $t>0$ only.) We prove this using duality. Recall that for $t>0$, and any $(n,m) \in \mathbb {N}_0^2$, $\mathbb {P}_{n,m}\big \{\tilde{N}(t) \in \{0,1\}\big \}=1$ and we can therefore calculate

$$\begin{aligned} \mathbb {E}[\tilde{\tilde{X}}(t)^n\tilde{ \tilde{Y}}(t)^m]&= x\mathbb {E}^{1,y}[{\bar{X}}^*(t)^n {\bar{Y}}^*(t)^m] + (1-x) \mathbb {E}^{0,y}[{\bar{X}}_*(t)^n {\bar{Y}}_*(t)^m]\\&= x\mathbb {E}^{1,y}[\tilde{X}(t)^n \tilde{Y}(t)^m] + (1-x) \mathbb {E}^{0,y}[\tilde{X}(t)^n \tilde{Y}(t)^m]\\&= x\mathbb {E}_{n,m}[1^{\tilde{N}(t)}y^{\tilde{M}(t)}] + (1-x)\mathbb {E}_{n,m}[0^{\tilde{N}(t)}y^{\tilde{M}(t)}]\\&= \mathbb {E}_{n,m}[(x + (1-x)1\mathrm {l}_{\{\tilde{N}(t)=0\}})y^{\tilde{M}(t)}]\\&= \mathbb {E}_{n,m}[x^{\tilde{N}(t)}y^{\tilde{M}(t)}] = \mathbb {E}^{x,y}[\tilde{X}(t)^n\tilde{Y}(t)^m]. \end{aligned}$$

Here, we used Proposition 3.8 in the second equality, the duality between $(\tilde{X}(t),\tilde{Y}(t))_{t \ge 0}$ and $(\tilde{N}(t),\tilde{M}(t))_{t \ge 0}$ from Theorem 3.3 in the third and last equality, and the observation, that $\mathbb {P}_{n,m}\big \{\tilde{N}(t) \in \{0,1\}\big \}=1$, in the fifth equality. Since $(n,m) \in \mathbb {N}_0^2$ was arbitrary, we have shown that for every $t>0$, $(\tilde{\tilde{X}}(t),\tilde{ \tilde{ Y}}(t))$ and $(\tilde{X}(t),\tilde{Y}(t))$ are equal in distribution. Since both processes are time-homogeneous Markov processes, this implies the claim. Thus, the process $({\hat{X}}(t), {\hat{Y}}(t))_{t \ge 0}$, defined as $({\hat{X}}(0),{\hat{Y}}(0)):=(x,y)$ and

$$\begin{aligned} \forall t\ge 0:\quad ({\hat{X}}(t),{\hat{Y}}(t)) := (\tilde{\tilde{X}}(t),\tilde{ \tilde{ Y}}(t)), \end{aligned}$$

is cádlág for all $t>0$ and coincides in distribution with $(\tilde{X}(t),\tilde{Y}(t))_{t \ge 0}$ started in $(\tilde{X}(0),\tilde{Y}(0))=(x,y)$. $\square$

#### Remark 3.9

(*Imbalanced Island size: Part 2*) We return to the example discussed in Remark 2.4 of the two-island model and its close relation to the seed bank model. The frequency process of the given allele is then described by the *two-island diffusion* (Kermany et al. 2008),

31 $$\begin{aligned} {\left\{ \begin{array}{ll} \mathrm{d} X(t) &{} = c(Y(t) -X(t))\mathrm{d}t + \alpha \sqrt{X(t)(1-X(t))}\mathrm{d}B(t), \\ \mathrm{d} Y(t) &{} = cK(X(t) -Y(t))\mathrm{d}t + \alpha '\sqrt{Y(t)(1-Y(t))}\mathrm{d}B'(t), \end{array}\right. } \end{aligned}$$

where $(B(t)_{t \ge 0}$ and $(B'(t))_{t \ge 0}$ are independent Brownian Motions.

Again, the interesting consideration here is to use *different* scalings of the coalescence rates in the islands, i.e. different scalings for $\alpha \ge 0$ and $\alpha '\ge 0$. If, in addition to $c\rightarrow 0$ we assume the coalescence rate $\alpha '>0$ in the second island to scale as *c*, i.e. $\alpha '/c \rightarrow 1$, the result is a two-island model with instantaneous coalescences in the first island, but otherwise regular migration and diffusive behaviour in the second. For more precision, denote by $(X^{c,\alpha '}(t), Y^{c,\alpha '}(t))_{t \ge 0}$ the *two-island diffusion* with migration rate $c>0$ and island 2 of size $\alpha '>0$ and assume that it starts at some $(x, y) \in [0,1]^2$, $\mathbb {P}$-a.s.. Repeating the calculations we did for the seed bank model, it can be shown that the sequence $(X^{c_{\kappa },\alpha '_{\kappa }}(t), Y^{c_{\kappa },\alpha '_{\kappa }}(t))_{t \ge 0}$ will converge to a Markovian degenerate limit coinciding in distribution with a Markov process with generator

$$\begin{aligned} \hat{ \mathcal L} f(x,y)&= (1-x)y(f(1,y)-f(x,y)) + x(1-y)(f(0,y)-f(x,y))\nonumber \\&\qquad + K(x-y)\frac{\partial f}{\partial y} (x,y) + \frac{1}{2}y(1-y)\frac{\partial ^2}{\partial y^2} f(x,y) \end{aligned}$$

for functions *f* in $\{f:\{0,1\}\times [0,1] \rightarrow \mathbb {R}\mid f(0, \cdot ), f(1,\cdot ) \in \mathcal C^2([0,1], \mathbb {R})$ whenever started in the smaller state-space $\{0,1\}\times [0,1]$.
